# Cytological, physiological, and transcriptomic analyses of golden leaf coloration in *Ginkgo biloba* L

**DOI:** 10.1038/s41438-018-0015-4

**Published:** 2018-03-01

**Authors:** Wei-xing Li, Shun-bo Yang, Zhaogeng Lu, Zhi-chong He, Yun-ling Ye, Bei-bei Zhao, Li Wang, Biao Jin

**Affiliations:** 1 College of Horticulture and Plant Protection, Yangzhou University, Yangzhou, 225009 China; 2 College of Resource and Environment, Xizang Agriculture and Animal Husbandry College, Tibet, 860000 China

## Abstract

*Ginkgo biloba* is grown worldwide as an ornamental plant for its golden leaf color. However, the regulatory mechanism of leaf coloration in *G. biloba* remains unclear. Here, we compared *G. biloba* gold-colored mutant leaves and normal green leaves in cytological, physiological and transcriptomic terms. We found that chloroplasts of the mutant were fewer and smaller, and exhibited ruptured thylakoid membranes, indistinct stromal lamellae and irregularly arranged vesicles. Physiological experiments also showed that the mutant had a lower chlorophyll, lower flavonoid and higher carotenoid contents (especially lutein). We further used transcriptomic sequencing to identify 116 differentially expressed genes (DEGs) and 46 transcription factors (TFs) involved in chloroplast development, chlorophyll metabolism, pigment biosynthesis and photosynthesis. Among these, the chlorophyll biosynthesis-related *PPO* showed down-regulation, while chlorophyll degradation-related *NYC/NOL* had up-regulated expression in mutant leaves. *Z-ISO*, *ZDS*, and *LCYE*, which are involved in carotenoid biosynthesis were up-regulated. Quantitative real-time PCR (RT-qPCR) further confirmed the altered expression levels of these genes at three stages. The alteration of *PPO* and *NYC*/*NOL* gene expression might affect chlorophyll biosynthesis and promote degradation of chlorophyll *b* to chlorophyll *a*, while the up-regulated genes *Z-ISO*, *ZDS* and *LCYE* enhanced carotenoid accumulation. Consequently, changes in the ratio of carotenoids to chlorophylls were the main factors driving the golden leaf coloration in the mutant *G. biloba*.

## Leaf color: Golden secrets of Ginkgo biloba

Studying a mutant variety of the popular ornamental tree *Ginkgo biloba* reveals insights into the molecular mechanisms controlling gold and yellow coloration in plant leaves. Biao Jin at Yangzhou University, China, and colleagues, compared the cell biology, physiology and gene activity of a gold-leafed mutant form of the tree and the normal green-leafed form. The golden mutant has fewer and smaller chloroplasts—the green bodies within cells that capture light energy during photosynthesis. These chloroplasts also have several disrupted internal structures. The levels of key pigment molecules, including chlorophylls, carotenoids and flavonoids are also altered in the mutant leaves. The most significant factor producing the golden coloration is an increased level of carotenoids relative to chlorophylls. Understanding the factors controlling leaf coloration might help horticulturists to manipulate this characteristic of ornamental trees and shrubs.

## Introduction

Leaf color is an important trait in ornamental plants, and leaf coloration has attracted widespread attention from both the public and researchers. The leaf color of higher plants is attributed to various leaf pigments, mainly chlorophyll, carotenoids and anthocyanin. The red leaves associated with anthocyanin have been extensively studied^1^. In contrast, despite being a common phenomenon, leaf yellowing has received less attention. Leaf yellowing is generally considered to be caused by chlorophyll breakdown unmasking yellow pigments, as chlorophyll dominates the major pigment content of normal green leaves. Accordingly, studies of leaf yellowing have generally focused on chlorophyll biosynthesis and degradation.

Because a series of enzymatic steps are involved in chlorophyll biosynthesis, blocking any step of chlorophyll synthesis in plants causes low chlorophyll content, resulting in green-deficient leaf color^2,3^. Many leaf color mutants with chlorophyll deficiencies have been studied^4–6^. Although leaf color mutants can function abnormally in terms of photosynthesis, they may also exhibit brighter non-green leaf coloration, which has esthetic value for ornamental plants. In fact, many cultivars with vivid foliage are propagated from such mutants, and the coloration mechanisms of leaf mutants have been studied at multiple levels. For example, the yellow leaf mutant of *Oryza sativa* exhibits abnormal chloroplasts in terms of morphology and distribution^7^. The yellow-striped leaves of a *Cymbidium sinense* mutant result from an increase in chlorophyll degradation and metabolism^6^. The yellow leaf mutation of *Lagerstroemia indica* is mainly due to differential expression of genes involved in chlorophyll biosynthesis and degradation^8^. Additionally, the yellow–white leaf mutant of *Camellia sinensis* utilizes genes related to metabolic pathways and carbon fixation for leaf coloration^9^.

*Ginkgo biloba* L. is known as one of the most ancient seed plants, and is thus referred to as a “living fossil”;^10^ it has been widely introduced around the world as an excellent landscape tree. The most attractive ornamental trait of *G. biloba* is its golden leaves in autumn. Despite the spectacular color, the mechanism underlying leaf coloration in *G. bilob*a remains poorly understood. In an early study of *G. biloba*, Matile et al^11^. reported that spectral optical properties change due to retention of carotenoids in yellow leaves in the autumn. Recently, Liu et al.^12^ analyzed the proteomic profiles of yellow and green *G. biloba* leaves and identified differentially accumulated proteins involved in energy metabolism, photosynthesis and carbon fixation. To better understand the mechanism of coloration and color variation in *G. biloba* leaves, it is important to compare yellow mutant leaves with green leaves under the same spatiotemporal and developmental conditions. Fortunately, our discovery of a novel golden-green striped mutant made such research possible.

In this study, we compared the golden–green striped mutant to normal green leaves in terms of structure, pigment content, chlorophyll synthesis precursors and transcriptomics. We identified differentially expressed genes (DEGs) and transcription factors (TFs) related to pigment biosynthesis and metabolism. Furthermore, we validated the expression of genes involved in leaf coloration using quantitative real-time polymerase chain reaction (RT-qPCR). Our results reveal the cytological, physiological and transcriptomic aspects of golden leaf coloration in *G. biloba* and provide a useful reference for leaf coloration studies of other plant species.

## Materials and methods

### Plant material

**Fig. 1:**
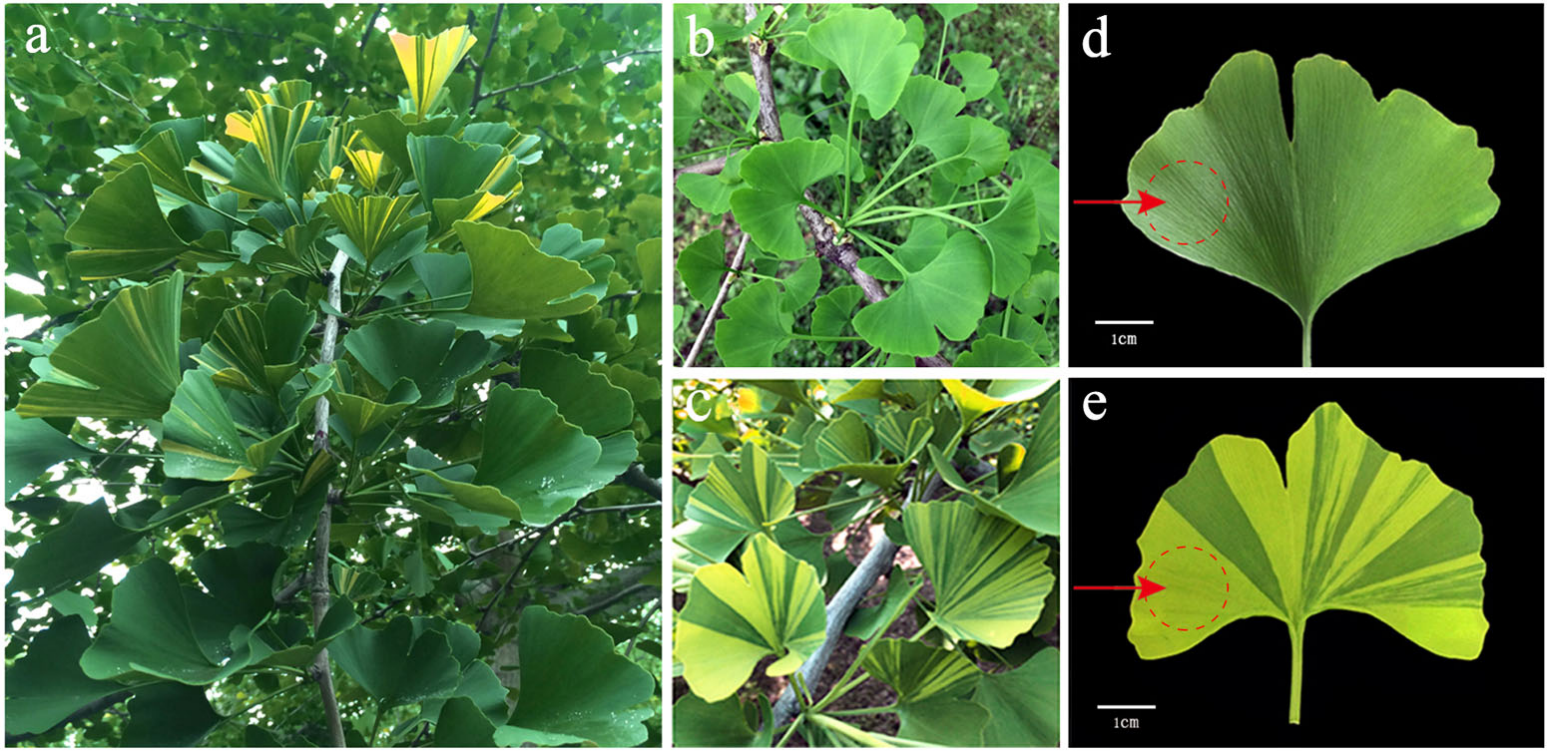
Phenotypes of normal green leaves and mutant leaves from the same short shoot of *Ginkgo biloba* in May. **a** Phenotype of the *G. biloba* tree. **b**, **d** Phenotype of the normal green leaf. **c**, **e** Phenotype of the mutant leaf. The red-dashed circles near arrows represent the sampling areas

A healthy adult *G. biloba* tree bearing both normal green leaves and golden–green striped leaves was used in this study, after growing under natural conditions in the *Ginkgo* nursery of Yangzhou University (32°39′ N, 119°42′ E). Normal green leaves and golden–green striped leaves borne on the same short shoot of this tree were collected from May to July in 2015 and 2016 (Fig. 1a–c). For cytological, physiological and RNA-Seq experiments, yellow parts of the golden–green striped leaves (mutant leaves) and the green leaves (normal leaves) were sampled separately in May. For RT-qPCR, the mutant leaves and green leaves from three stages (May to July) were used. All of the samples were collected from the identical spatial sections of leaves (Fig. 1d, e), and immediately frozen in liquid nitrogen, and stored at −80 °C until use.

### Measurements of chlorophyll, carotenoid, and flavonoid contents

Approximately 0.1 g of leaves from normal green leaves and the golden parts of mutant leaves (sampled in May) were cut into pieces and submerged in 80% acetone overnight to extract the chlorophyll. Next, the extract was measured spectrophotometrically at 665, 649, and 470 nm with a UV-1800 spectrophotometer (DU-800; Beckman Coulter, Brea, CA, USA). Soil-plant analysis development (SPAD) values were measured in situ on each type of leaf using a Chlorophyll Meter SPAD-502Plus (Konica Minolta, Osaka, Japan). To measure the contents of chlorophyll intermediaries, leaves were homogenized in nine volumes of 0.01 M phosphate-buffered saline, mixed on ice, and centrifuged (30 min at 2500 g). The supernatant was assayed separately with an ELISA kit (HengYuan Biological Technology Co., Ltd, Shanghai, China). Carotenoid components were measured using high-performance liquid chromatography, and total carotenoid and flavonoid contents were determined using a UV-1800 spectrophotometer. Three biological replicates were evaluated for each sample. The data thus obtained were analyzed using SPSS software (ver. 17.0; SPSS Inc., Chicago, IL, USA).

### Transmission electron microscopic observation

Samples dissected from normal green leaves and the golden parts of mutant leaves were cut into smaller sections approximately 0.5 × 0.5 × 0.5 mm in size. After pre-fixation in 4% glutaraldehyde for 24 h at 4 °C, followed by 1% OsO_4_ for 2 h, the tissues were dehydrated through an acetone series. For ultrastructural observations, 70-nm thick sections were cut with a Leica EM UC6 ultramicrotome (Leica Microsystems GmbH, Wetzlar, Germany), and stained with 1% (w/v) uranyl acetate and 1% (w/v) lead citrate. Leaf cells were observed and photographed using a Philips Tecnai 12 transmission electron microscope (JEOL Ltd., Tokyo, Japan). The chloroplasts in 30 randomly selected cells were counted using the ultrastructural images, and the average number of chloroplasts per cell was calculated. The mean size of chloroplasts in 30 intact chloroplasts distributed in different cells was determined using AutoCAD. The data obtained were analyzed using the SPSS software.

### RNA extraction and preparation of cDNA library

Total RNA was isolated from normal green leaves and the golden parts of mutant leaves using the MiniBEST Plant RNA Extraction Kit (TaKaRa, Dalian, China) (Fig. 1d, e). Two biological replicates were analyzed for each RNA sample. The quantity and quality of each RNA sample were assessed using 1% agarose gel electrophoresis and examined with a Nanodrop 1000 spectrophotometer (Nanodrop, Wilmington, DE, USA). RNA integrity and concentration were checked using an Agilent 2100 Bioanalyzer (Agilent Technologies, Inc., Santa Clara, CA, USA). Briefly, mRNA was first isolated using the NEBNext Poly (A) mRNA Magnetic Isolation Module (E7490; NEB, Ipswich, MA, USA). Then, the enriched and purified mRNA was broken into short RNA inserts of approximately 200 nt, which were used to synthesize the first and second strands of cDNA. Next, the double-stranded cDNA were subjected to end-repair, dA-tailing and adaptor ligation. Finally, suitable fragments were isolated and then enriched using polymerase chain reaction (PCR) amplification.

### Illumina deep sequencing and data analysis

The cDNA libraries constructed from the samples were sequenced on a flow cell using the Illumina HiSeq™ 4000 sequencing platform (Illumina, San Diego, CA, USA). cDNA library construction and sequencing were performed by Beijing Biomarker Biotechnology Co. Ltd (Beijing, China). From the raw reads, low quality reads, such as adaptor sequences, and reads with >10% unknown nucleotides were removed using a Perl script. The clean reads, which were filtered from the raw reads, were mapped to a *G. biloba* reference genome (http://gigadb.org/dataset/100209) using HISAT v2.0.4 software with default parameters. All clean data were deposited in the NCBI Sequence Read Archive. The read counts for each gene were calculated with HTSeq v0.6.1, and gene expression levels were estimated as fragments per transcript kilobase per million fragments mapped (FPKM) values. All DEG data sets are available at the NCBI SRA under accession number GSE103827.

### Identification and functional analysis of DEGs

To identify genes that were differently expressed between the libraries created from normal green leaves and gold-colored mutant leaves, the DESeq R package (ver. 1.18.0) was used in this study. DESeq provides statistical routines for identifying differential expression in gene expression data using a model based on the negative binomial distribution. To control for the false discovery rate, *P*-values obtained from DESeq were adjusted using the Benjamini approach^13^. Genes with an adjusted *P*-value < 0.05 were considered differentially expressed. The DEGs identified were used for Gene Ontology (GO) and Kyoto Encyclopedia of Genes and Genomes (KEGG) enrichment analysis. GO enrichment analysis of DEGs was performed using the GOseq R package. GO terms with corrected *P*-values < 0.05 were considered significantly enriched among differently expressed genes. To further elucidate the biological functions of DEGs, they were assigned to the diverse pathways of the KEGG database. Then, KOBAS software was used to test the statistical enrichment of DEGs within KEGG pathways. Annotations of DEGs were retrieved from the *Ginkgo biloba* Genome Annotation Project. Venny software (http://bioinfogp.cnb.csic.es/tools/venny/index.html) was used to identify overlapping DEGs among different samples.

### RT-qPCR analysis

The RT-qPCR experiments were used to confirm and analyze the basic expression levels of a subset of candidate genes. Total RNA was isolated from normal green leaf and gold-colored mutant leaf samples collected in May, June and July, as described above. Each RNA sample was treated with gDNA Eraser following the manufacturer’s instructions to eliminate any contaminant gDNA, using 1 μg of total RNA. The treated RNA solution (10 μL) was subjected to reverse transcriptase reactions via PrimeScript™ Reverse Transcriptase Reagent Kit with gDNA Eraser (Perfect Real Time; TaKaRa) in accordance with the manufacturer’s protocol. Gene-specific primers were designed using Primer 5.0 (Supplementary Table S4). The *G. biloba* gene GAPDH was used as an internal reference gene. RT-qPCR was performed using the CFX96™ Real-Time System (Bio-Rad, Hercules, CA, USA) with the SYBR Premix Ex Taq™ Kit (Perfect Real Time; TaKaRa) in accordance with the manufacturer’s protocol. RT-qPCR conditions were as follows: 30 s at 95 °C for denaturation, followed by 40 cycles of 5 s at 95 °C, 30 s at 55 °C, and 10 s at 72 °C. All reactions were performed in three biological replicates, and the resultant threshold cycle (Ct) values were determined using Bio-Rad CFX Manager software (ver. 1.6.541.1028). Relative expression levels of target genes were calculated with the 2^−ΔΔCt^ comparative Ct method^14^.

## Results

### Cytological changes in mutant leaves

The gold-colored leaf mutant of *G. biloba* was obtained through normal growth on the same plant and branch as green leaves due to a bud mutation. At the beginning of the growth period in April, the mutant leaves exhibited no difference from normal green leaves. Then, the mutant gradually differentiated into golden-striped leaves from May to August, while the normal leaves of *G. biloba* remained green. The normal green leaves and the green stripes of the golden mutant leaves gradually turned yellow in September and were completely yellow by late November to early December.

**Fig. 2:**
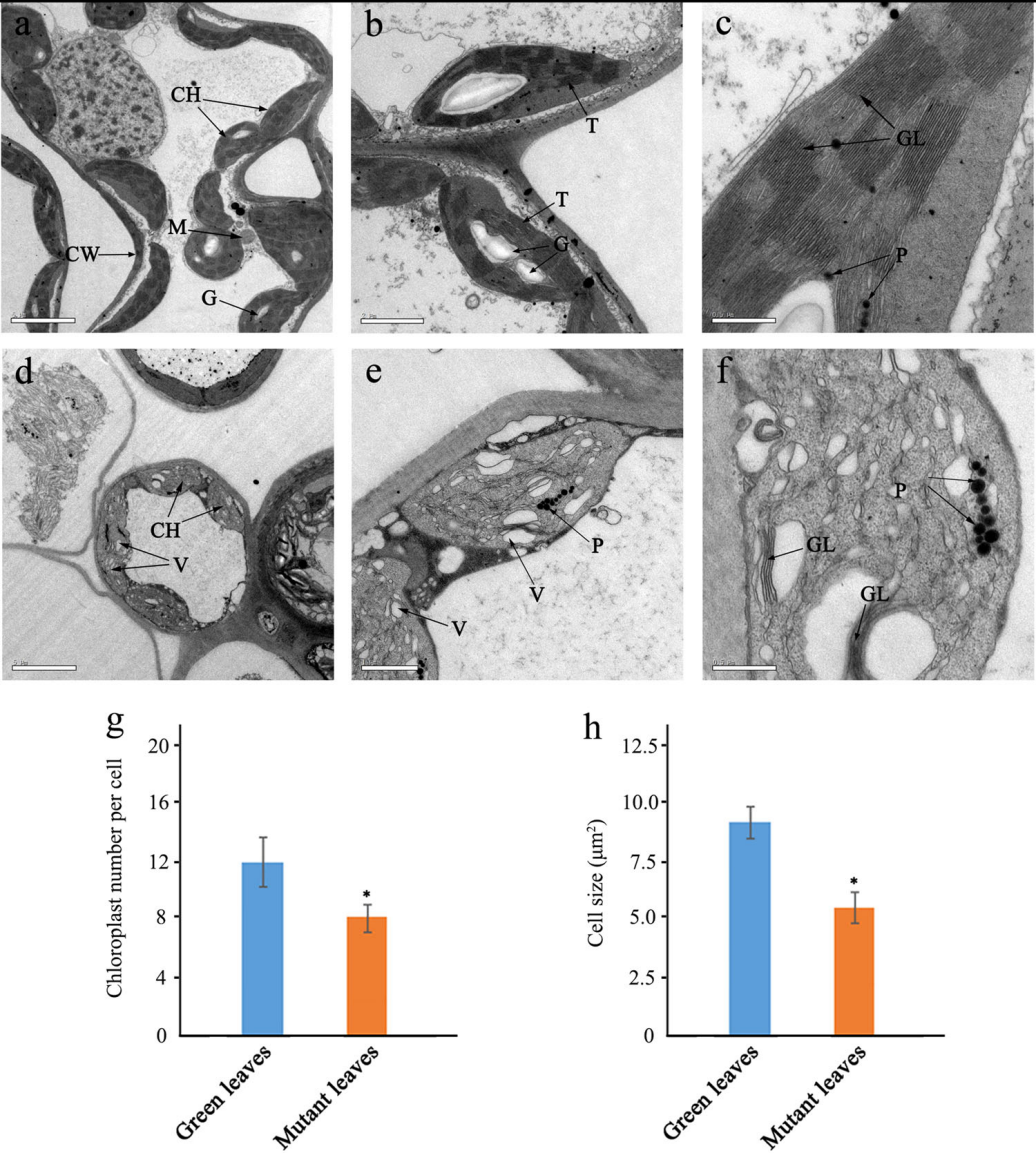
Chloroplast ultrastructure on a normal green leaf and mutant leaves in *G. biloba*. **a**, **b**, **c** Chloroplast ultrastructures showing typical structures and distinct thylakoid membranes in the normal green leaf. **d**, **e**, **f** Abnormal chloroplast ultrastructures contained irregularly arranged vesicles filled with numerous plastoglobuli in the mutant. Bars = 5 μm (**a**, **d**), 2 μm (**b**), 1 μm (**e**), 0.5 μm (**c**, **f**). CH chloroplast, CW cell wall, M mitochondria, V vacuole, P plastoglobuli, T thylakoid grana, G granulose, GL grana lamella (**g**, **h**) The average number of chloroplasts per cell and mean chloroplast size were lower in the mutant leaves

We further compared the ultrastructure of chloroplasts in normal green leaves and the golden parts of mutant leaves. In the mesophyll cells of normal green leaves, chloroplasts showed typical structures, with small starch granules and few plastoglobuli (Fig. 2a, b) with distinct thylakoid membranes and stromal lamellae (Fig. 2c). In contrast, ultrastructural analysis of chloroplasts in the mutant leaves revealed ruptured thylakoid membranes, indistinct or absent stromal lamellae, and some chloroplasts containing irregularly arranged vesicles (Fig. 2d, e). Furthermore, in the mutant leaves, chloroplasts were crowded with a large number of vesicles and filled with numerous plastoglobuli (Fig. 2f). Additionally, the average number of chloroplasts per cell and mean chloroplast size in the mutant leaves were significantly lower than those of normal green leaves (Fig. 2g, h).

### Physiological changes in the mutant leaves

**Fig. 3:**
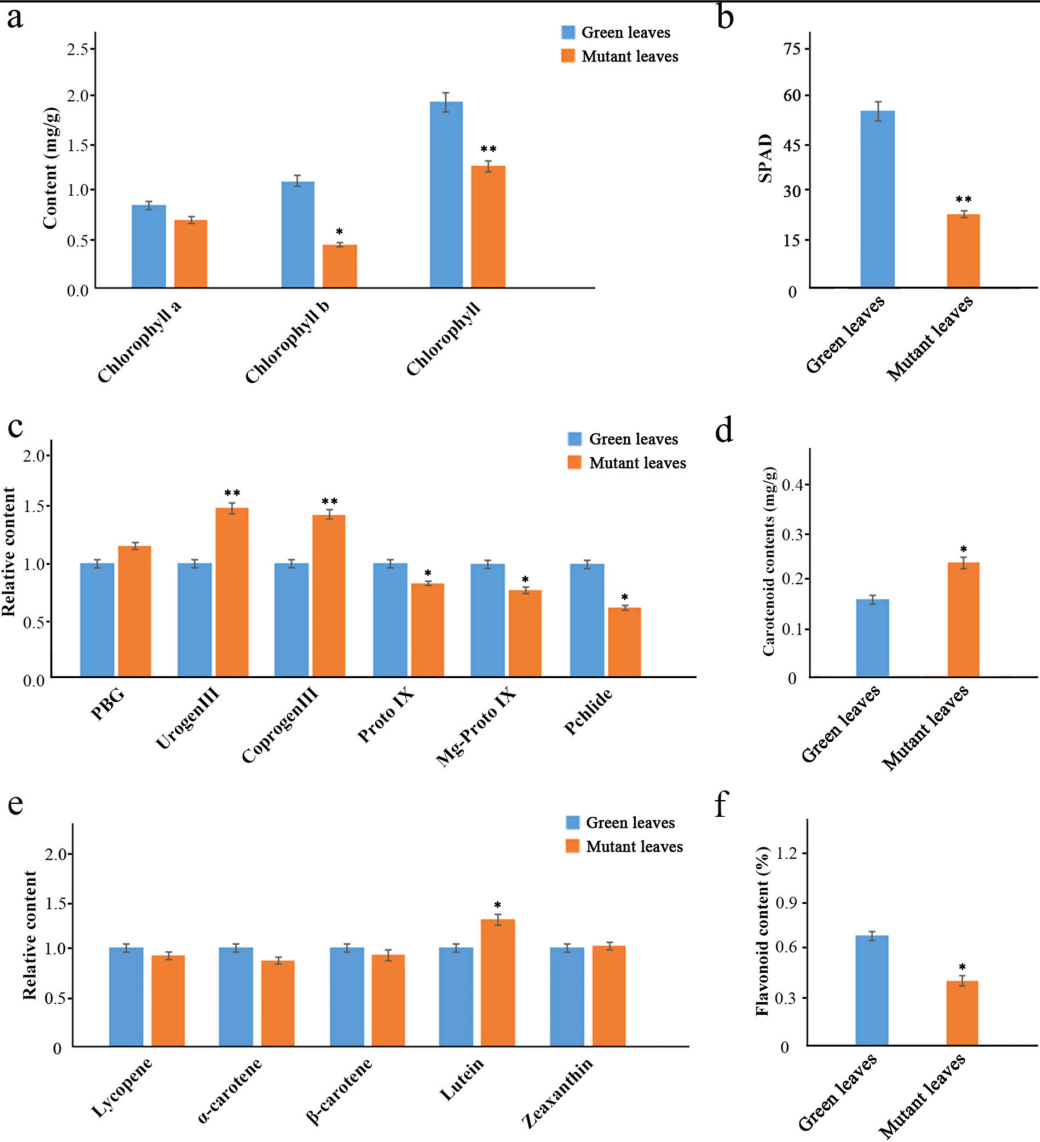
Determination of pigment contents in *G. biloba*. **a** Chlorophyll contents of normal green and mutant leaves **b** Soil-plant analysis development (SPAD) values of normal green and mutant leaves. **c** Relative content of chlorophyll intermediaries between normal green and mutant leaves. **d** Total carotenoid contents of normal green and mutant leaves. **e** Relative content of carotenoid components between normal green and mutant leaves. **f** Total flavonoid contents of normal green and mutant leaves. Asterisks indicate: (*) *P* ⩽ 0.05, (**) *P* ⩽ 0.01

In parallel with the evident cytological changes in mutant leaves, we analyzed changes in the pigment contents of normal green leaves and mutant leaves. Compared with the normal leaves, the mutant leaves showed an 18.4% decrease in chlorophyll *a* content and a 59.5% decrease in chlorophyll *b* content (Fig. 3a). We further calculated the SPAD value, and found that in normal green leaves it was 57.8, i.e., greater than the 24.3 of the mutant, indicating abnormal or delayed metabolism of chlorophyll in the mutant leaves of *G. biloba* (Fig. 3b). Further detailed analysis showed that the chlorophyll synthesis precursor porphobilinogen, uroporphyrin III (Urogen III) and coproporphyrin III (Coprogen III) contents increased significantly, by about 10–20%, in the mutant leaves. On the other hand, the protoporphyrin IX (Proto IX), magnesium protoporphyrin (Mg-Proto) and protochlorophyllide (Pchlide) contents decreased markedly, by about 10–15%, in the mutant leaves compared with normal green leaves (Fig. 3c). In addition, the total carotenoid content of mutant leaves was 0.25 mg/g, which was higher than that of normal green leaves (0.17 mg/g; Fig. 3d). Then, we measured the major carotenoid components in our samples. The lutein content of the mutant was significantly higher than that of the green leaves, whereas we detected no significant differences among the other carotenoid components (Fig. 3e). The ratio of carotenoids to chlorophylls in the mutant (approximately 0.21) was two-fold higher than that in green leaves (approximately 0.10). Additionally, the total flavonoid contents in the mutant were lower than those in the green leaves (Fig. 3f).

### Transcriptomic alterations in mutant leaves

Based on the phenotypes described above, as well as ultrastructural and physiological changes, we speculated that the expression pattern of genes responsible for chloroplast development and division, as well as pigment biosynthesis, had been altered in mutants. To test our hypothesis, a transcriptomic comparison was carried out. In this comparison, approximately 5.81–8.12 GB of clean bases were produced using an Illumina Hi-Seq^TM^ 4000 sequencer. After stringent quality checks and data cleanup, 39.2–54.9 million clean reads were obtained through sequencing of the cDNA libraries prepared from normal green leaves and mutant leaves. The clean sequence data were deposited into the Short Read Archive database of the NCBI. Then, a total of 37.0–51.7 million reads were mapped to the *G. biloba* genomic database, with match ratios in the range of 94.16–94.69% (Table S1).

FPKM methods were used to analyze gene expression patterns in both types of library. When comparing the two types of library with respect to the FPKM calculation, 20,442 and 20,225 genes were identified in the cDNA libraries, while 1068 and 851 genes were expressed specifically in normal green leaves and mutant leaves, respectively (Figure S1). A total of 5235 DEGs (*P*-value < 0.01, |fold-change| > 2) were detected, including 2933 up-regulated genes and 2302 down-regulated genes (Figure S2). The functions of these DEGs were classified according to the GO database using the Blast2GO software suite. Up-regulated DEGs were enriched in the GO terms “catalytic activity” and “single-organism process”, while down-regulated DEGs were enriched in the “carbohydrate phosphatase activity” and “RNA metabolic process” terms (Figure S3). In addition, all DEGs were matched and assigned to 116 KEGG pathways. The main enriched pathways were “Biosynthesis of secondary metabolites,” “Phenylalanine metabolism,” and “Porphyrin and chlorophyll metabolism” (Figure S4a). In mutant leaves, many down-regulated DEGs were enriched in “Biosynthesis of secondary metabolites,” whereas some up-regulated DEGs were enriched in “Porphyrin and chlorophyll metabolism” and “Carotenoids biosynthesis” pathways (Figure S4b, c; red arrow).

### Genes regulating chlorophyll development and metabolism are involved in leaf coloration

Chloroplast development and division plays an important role in the coloration of plant leaves. We identified DEGs regulating chloroplast development and division in transcriptomic data based on KEGG pathway annotations. The *GLK* (Golden 2-like) gene family acts as a regulatory factor for chloroplast development, and *Ftsz* genes are involved in cell division. Compared with normal green leaves, the *GLK1* gene showed down-regulated expression, and the expression levels of *Ftsz* genes were similarly reduced in the mutant (Table S2).

**Fig. 4:**
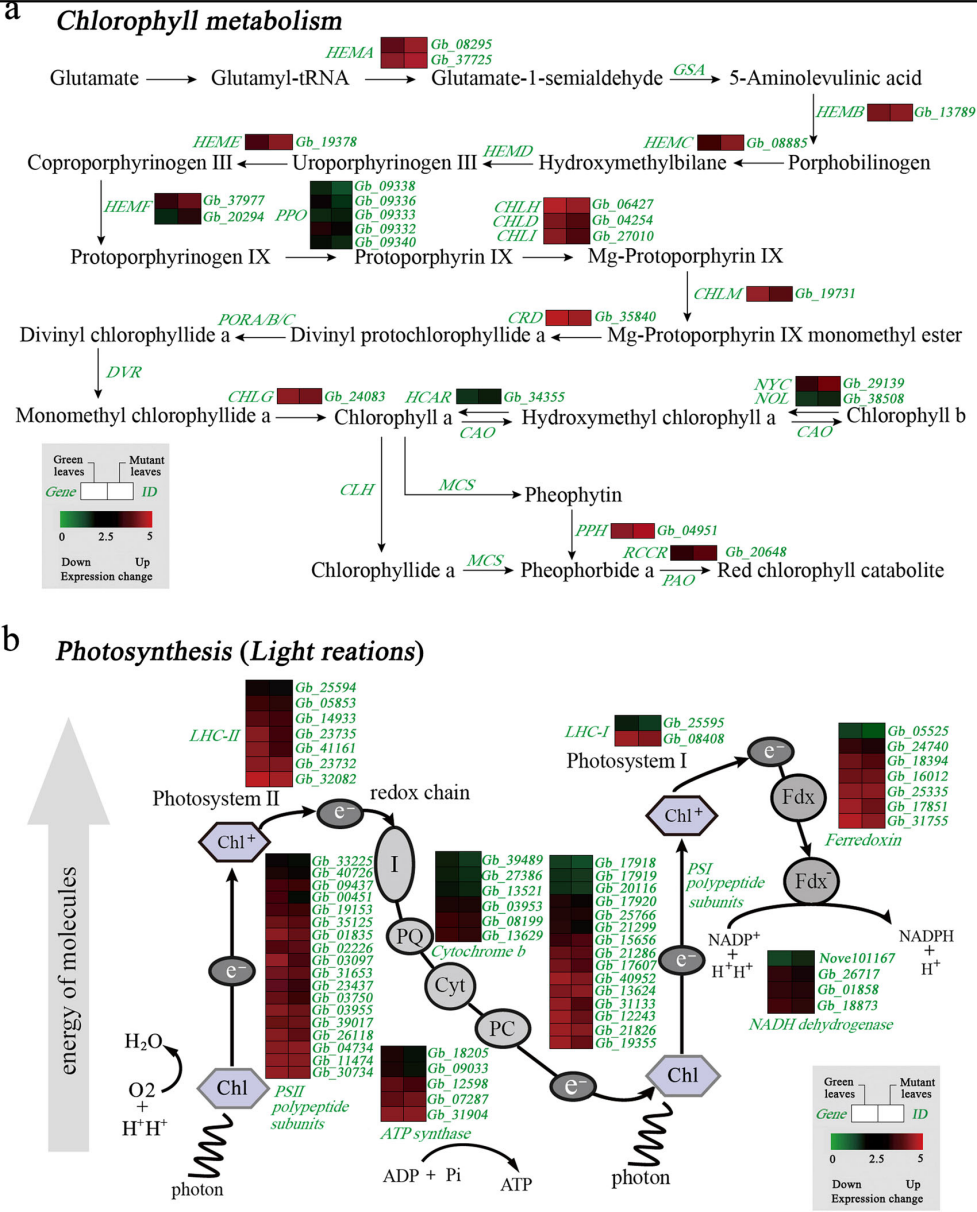
**a** Expression profiles of differentially expressed genes (DEGs) involved in chlorophyll biosynthesis and degradation between the normal green leaf and the mutant in *G. biloba*. **b** Expression profiles of DEGs involved in photosynthesis between the normal green leaf and the mutant in *G. biloba*

Chlorophylls are essential to photosynthesis, light harvesting, and energy transduction. The chlorophyll metabolic pathway always includes the following three phases: biosynthesis of chlorophyll *a*, interconversion between chlorophylls *a* and *b*, and degradation of chlorophyll *a*^15,16^. In our study, 18 DEGs related to chlorophyll biosynthesis and five DEGs associated with chlorophyll degradation were identified based on KEGG pathway assignments (Table S2). The expression levels of DEGs were determined via hierarchical cluster analysis (Fig. 4a). Five *PPO* genes were identified, most of which were significantly down-regulated in the mutant, suggesting that decreased expression of these genes might contribute to the low biosynthetic efficiency of Proto IX. Moreover, *CHLH*, *CHLD* and *CHLI*, which are subunits of Mg-chelatase, also had reduced mRNA levels in the mutant, while two *NYC1/NOL* genes, which are closely related to chlorophyll degradation, were significantly up-regulated in the mutant. This result indicates that chlorophyll degradation genes were expressed in the leaf color mutant at a significantly higher level than in green leaves.

Compared to normal green leaves, the expression levels of DEGs related to photosynthesis were significantly down-regulated in the mutant (Fig. 4b). For example, genes in the LHCA and LHCB families, which are thought to bind chlorophyll *a* and chlorophyll *b*, and transfer excitation energy to the reaction centers in photosystem I (IPSI) and photosystem II (PSII), showed very significant reductions in mRNA levels in the mutant. Furthermore, 15 DEGs related to the PSI reaction center subunit, and 18 related to the PSII reaction center subunit, showed reduced expression levels in the mutant compared with normal green leaves.

### Carotenoid and flavonoid biosynthesis genes involved in leaf coloration

**Fig. 5:**
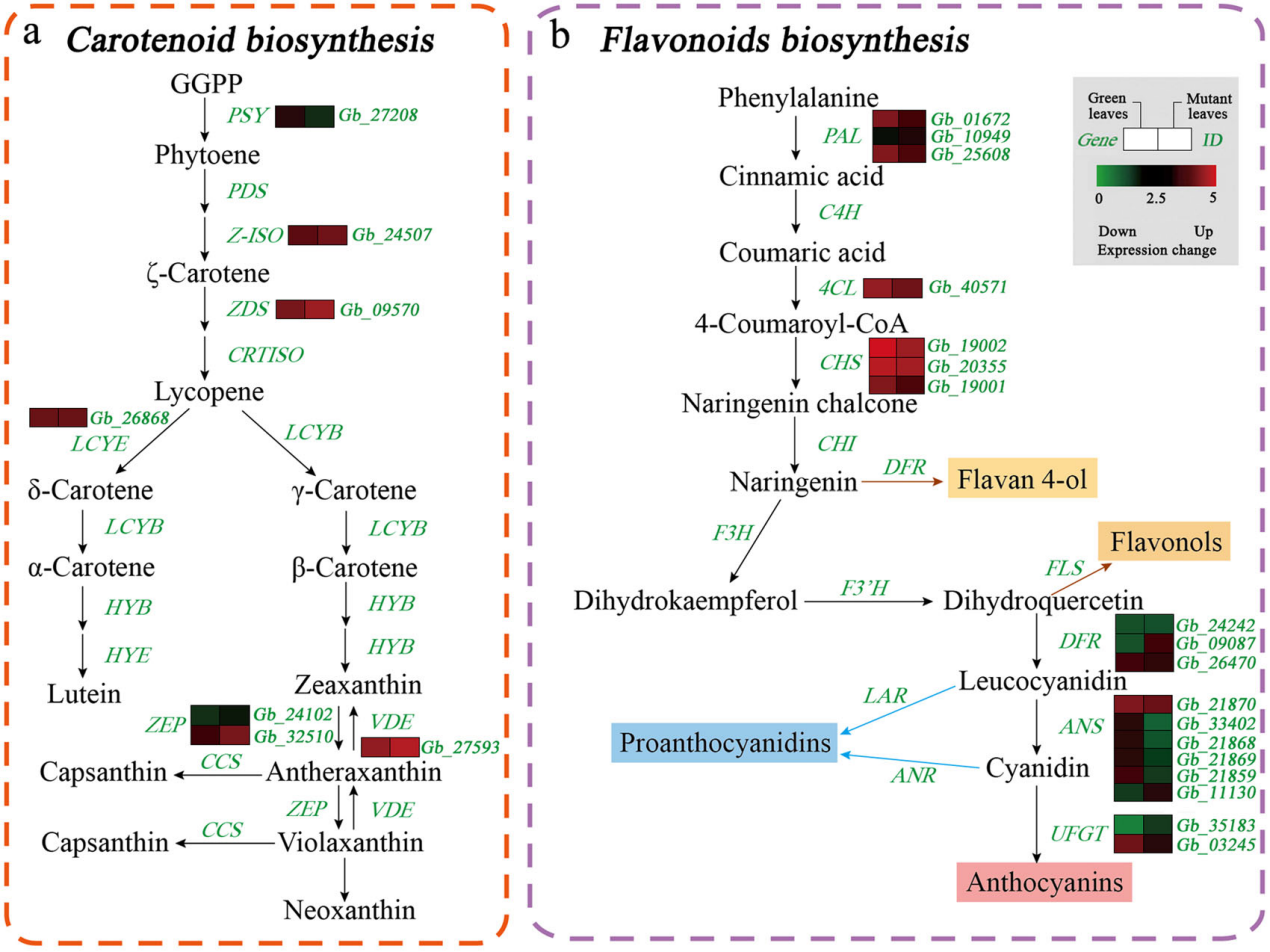
Expression profiles of DEGs associated with pigment biosynthesis between normal green leaves and mutant leaves in *G. biloba*. **a** Expression profiles of DEGs involved in carotenoid biosynthesis. **b** Expression profiles of DEGs involved in flavonoid biosynthesis

In our dataset, seven DEGs were annotated as key genes encoding enzymes related to carotenoid biosynthesis. In the mutant, some DEGs encoding Z-ISO, ZDS, LCYE and ZEP were significantly up-regulated with the exception *PSY* (Fig. 5a, Table S3).

We further identified 18 DEGs associated with flavonoid biosynthesis (Fig. 5b). The expression of 14 of these DEGs was down-regulated in the mutant, including *PAL*, *4CL*, *CHS*, *DFR*, *ANS* and *UFGT*.

### TFs involved in leaf coloration

**Fig. 6:**
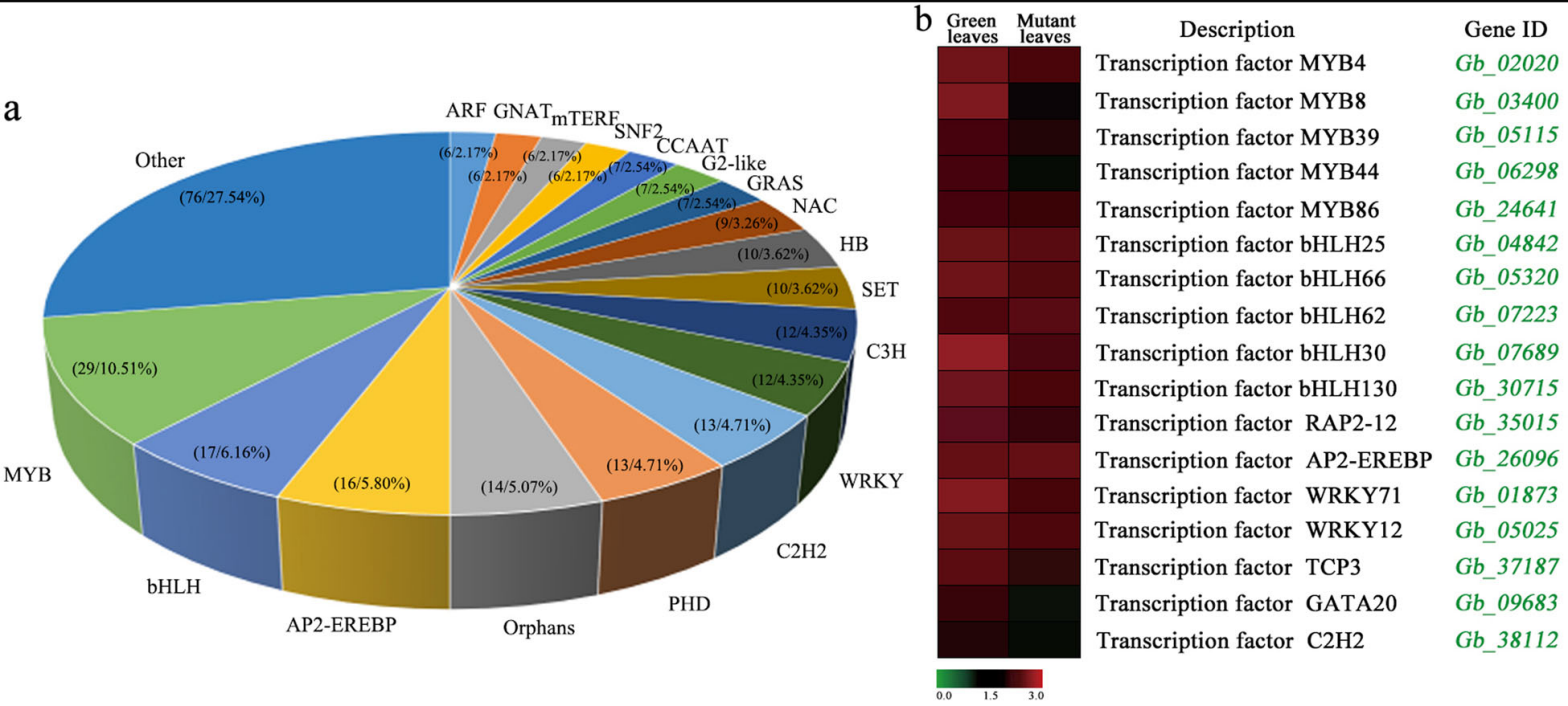
**a** Analysis of transcription factors (TFs) in normal green leaves and mutant leaves in *G. biloba*. **b** Expression profiles of TFs in normal green leaves and mutant leaves

TFs are key regulatory proteins that play important roles in regulating gene expression in various plant biological processes. In our dataset, we found that 276 DEGs were putatively identified as TFs associated with 56 TF families. The most abundant TF family was the MYB superfamily (29, 10.51%), followed by bHLH (17, 6.16%), AP2-EREBP (16, 5.80%), orphans (14, 5.07%) and WRKY (12, 4.35%; Fig. 6a). The MYB family was represented by 24 DEGs that were down-regulated in the mutant. A total of 17 DEGs associated with bHLH TFs were identified, and most exhibited highly significant down-regulation in the mutant. Additionally, some DEGs encoding AP2-EREBP, WRKY, TCP and C2H2 family TFs also showed significantly lower expression in the mutant compared to normal green leaves (Fig. 6b).

### Validation by RT-qPCR of some DEGs

**Fig. 7:**
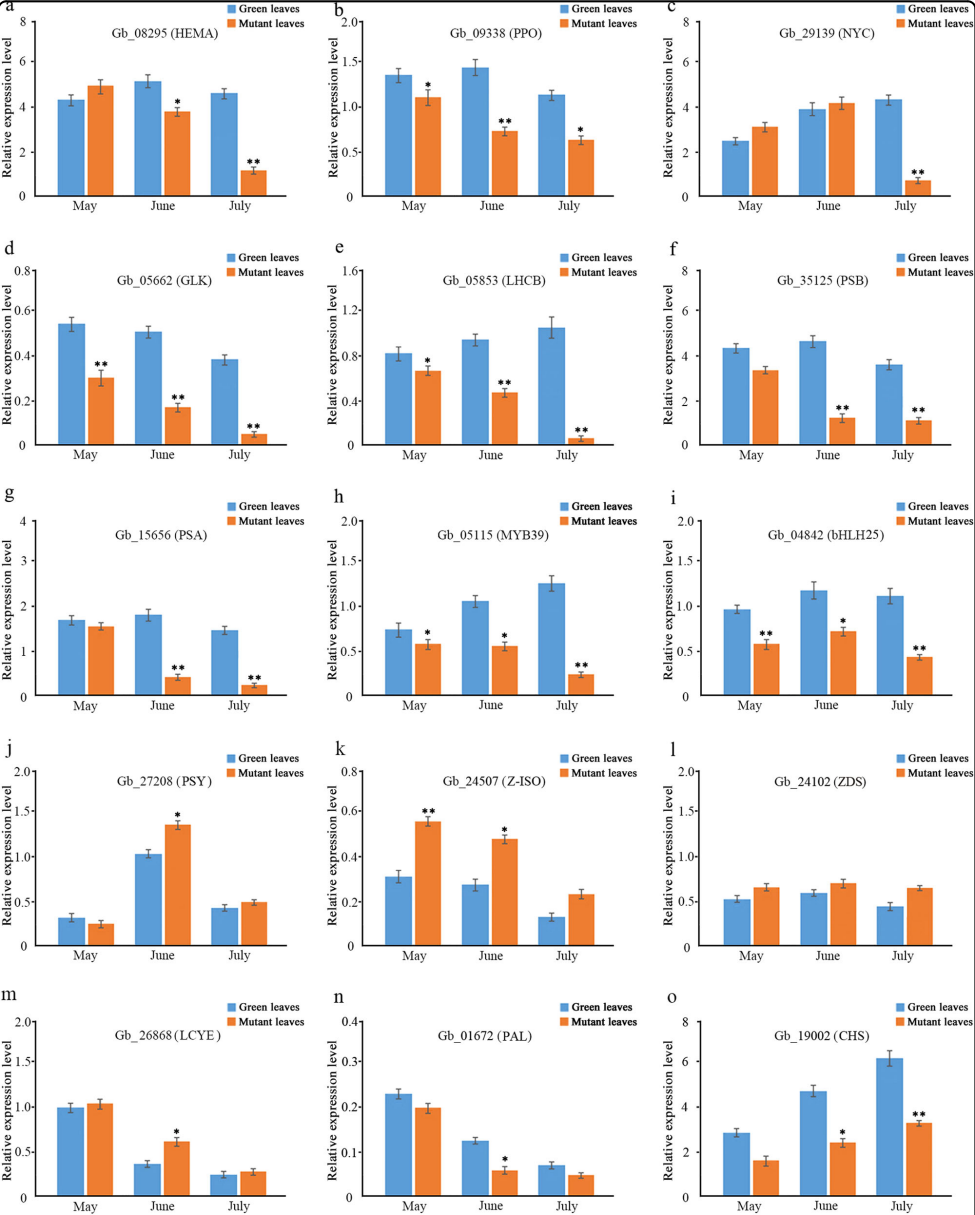
Quantitative real-time PCR (RT-qPCR) analysis of the expression of fifteen DEGs at different developmental stages between normal green leaves and mutant leaves in *G. biloba*. (**a**, **b**, **c**) DEGs involved in chlorophyll metabolism (**d**) DEGs involved in chloroplast development (**e**, **f**, **g**) DEGs involved in photosynthesis (**h**, **i**) DEGs involved in TFs regulating flavonoid biosynthesis (**j**, **k**, **l**, **m**) DEGs involved in carotenoid biosynthesis (**n**, **o**) DEGs involved in flavonoid biosynthesis. Asterisks indicate: (*) *P* ⩽ 0.05, (**) *P* ⩽ 0.01

To confirm the changes in expression of genes related to chlorophyll biosynthesis and degradation, photosynthesis and flavonoid biosynthesis, we performed differential expression analysis of the identified DEGs and TFs at three different developmental stages. The expression of nine DEGs was detected, including three genes involved in chlorophyll biosynthesis and degradation, one gene involved in chlorophyll development, three genes involved in photosynthesis and two TFs involved in flavonoid biosynthesis. The expression levels of the nine genes detected via RT-qPCR showed a pattern similar to that observed in the transcriptomic data (Fig. 7). Moreover, some DEGs showed significantly lower expression levels in the mutant over different developmental stages in May, June and July, such as *GLK* and *LHCB* (Fig. 7d, e). Additionally, *HEMA* genes, which are involved in chlorophyll biosynthesis, were up-regulated in May, but down-regulated in June and July, while *PPO* genes were down-regulated in the mutant compared to normal green leaves during three developmental stages (Fig. 7a, b). At the same time, the gene encoding *NYC*, which is involved in chlorophyll degradation, was up-regulated in May and June, but down-regulated in July. To examine more important gene expression profiles involved in carotenoid and flavonoid biosynthesis, we investigated four important genes for carotenoid biosynthesis and two important genes for flavonoid biosynthesis. In carotenoid biosynthesis, the gene expression of *PSY* was down-regulated in the mutant in May (Fig. 7j) and then up-regulated in June and July, whereas the other genes such as *Z-ISO*, *ZDS* and *LCYE* were up-regulated in three stages (Fig. 7k, l, m). In flavonoid biosynthesis, expression of the *PAL* and *CHS* genes was down-regulated in the mutant in three stages (Fig. 7n, o). Two differentially expressed TFs (MYB39 and bHLH25), which affect flavonoid synthesis in *G. biloba*, showed lower expression levels in the mutant at three developmental stages (Fig. 7h, i). In general, the qPCR results were consistent with the RNA-Seq data, despite some differences in expression levels.

## Discussion

### Chlorophyll pigment content, proportion and chloroplast development

Leaf color is an important commercial trait for ornamental plants. In higher plants, leaf coloration depends on chloroplast development and division, and chlorophyll biosynthesis and transport;^17^ thus, leaf color mutations are usually chlorophyll-deficiency mutations. Many chlorophyll-deficient mutants have been found in *Zea mays*, *O. sativa* and *Arabidopsis thaliana*^18–20^. In our study, we identified the novel chlorophyll-deficient chlorina mutant of *G. biloba*, which presents a golden-green striped phenotype. The chlorophyll *b* content in the mutant leaves is significantly lower than that of normal green leaves, suggesting that a lack of chlorophyll *b* decreases the total chlorophyll content and contributes to leaf color variation at the physiological level.

In chloroplasts, thylakoid membranes are arranged regularly and stacked into grana. The presence of grana stacks indicates that compact, light-harvesting machinery is extremely efficient in the absorption and conversion of light energy^21^. Previous studies have reported that most leaf color mutants show some alteration in the structural properties of thylakoid membranes^22^. For example, in bamboo leaf tissues, all chloroplasts in green leaves had abundant thylakoid membranes, while thylakoid membranes were converted into numerous abnormal vesicles in albino leaves^23^. Similarly, in normal mesophyll cells of *Anthurium andraeanum*, the chloroplasts have a typical structure containing small starch granules, while accumulation of large starch granules led to large gaps among stroma thylakoids in the leaf mutant^17^. In our study, we also found that the structure of chloroplasts in mutants differed significantly from that of normal green leaves. The ultrastructure of chloroplasts from mutants was severely altered, with some chloroplasts containing ruptured thylakoid membranes that were crowded by vesicles, lacked inner member structures, and were filled with numerous plastoglobuli, thus suggesting abnormal development of chloroplasts in this mutant. Moreover, the number and size of chloroplasts both decreased in mutant leaves. Therefore, leaf color changes in mutants might reflect abnormal development and function of plastids in the mutated leaves.

### RNA-Seq analysis and the DEGs involved in chlorophyll accumulation

Illumina sequencing and transcriptomic profiling provide information on quantitative changes in gene expression^24,25^. Thus, RNA-seq technology coupled with improved analysis methods enables recognition of novel transcript isoforms and their putative roles in leaf coloration. In the present study, transcriptomic profile analysis comparing normal green leaves with those of the gold-colored mutant revealed a number of DEGs. In total, 5,235 DEGs were identified through mapping to a reference genome. The number of DEGs is greater than 858 in leaf color mutants of *A. andraeanum*^17^ and less than 8,790 among three color developmental stages in *C. sinensis*^9^. Moreover, it is worth noting that some DEGs in our dataset related to chlorophyll biosynthesis and degradation, chloroplast development, photosynthesis and pigment biosynthesis are likely involved in leaf coloration in *G. biloba*.

Chlorophylls are responsible for harvesting and transferring solar energy in antenna systems, and for charge separation and electron transport in reaction centers^26^. In *A. thaliana*, 27 genes encode 15 enzymes involved in chlorophyll biosynthesis^27^. Changes in the expression of these genes might result in chlorophyll metabolic disorders and lead to a yellowing phenotype in plants. The *HEMA* gene encodes glutamyl-tRNA reductase (GluTR), which catalyzes the initial enzymatic step of tetrapyrrole biosynthesis in plastids, which eventually leads to chlorophyll production^28^. *HEMA* expression level changes might result in decreased chlorophyll biosynthesis and reduced chlorophyll content in an *L. indica* yellow leaf mutant^16^. Protoporphyrinogen IX oxidase (PPO) catalyzes the oxidation of protoporphyrinogen IX to Proto IX and also plays an important role in the process of chlorophyll synthesis. Inhibition of *PPO* leads to non-enzymatic formation of Proto IX. Uncoupling of Proto IX generation and iron insertion by ferrochelatase leads to a loss of feedback control over chlorophyll biosynthesis^29^. Moreover, Mg-chelatase deficiency is a common factor among many chlorophyll-deficient mutants. This enzyme complex consists of three subunits, *ChlD*, *ChlH* and *ChlI*. In transcriptomic research on the yellow-leaf tea cultivar of *C. sinensis*, expression of these three genes disrupted assembly of the enzyme complex and consequently influenced Mg-chelatase activity^5^. In our study, a total of 17 DEGs related to chlorophyll biosynthesis were identified based on KEGG pathway assignments. Among these DEGs, *GbHEMA* showed greatly enhanced mRNA levels in the leaf color mutant, similar to findings from the leaf color mutant of *C. sinensis*^5^. However, expression levels of *GbPPO* were highly significantly reduced compared with those in normal green leaves, which was further confirmed through RT-qPCR, thus suggesting that a late stage of chlorophyll biosynthesis was blocked. Parallel experiments also showed that the content of Coprogen III was about 25% higher, while the content of Proto IX was about 30% lower in the mutant compared to green leaves. Since our physiological results also show changes in chlorophyll precursor contents, and our transcriptomic analysis found that the expression levels of genes changed at this step, it is reasonable to conclude that there is an obstacle between protoporphyrinogen IX and Mg-Proto IX during the process of chlorophyll biosynthesis. Moreover, in this study, expression of three Mg-chelatase subunits, *GbCHLD*, *GbCHLH* and *GbCHLI*, showed a lower level in the mutant than in normal leaves, which might cause the low efficiency of chlorophyll biosynthesis. This result was further confirmed by the reduced contents of chlorophylls *a* and *b*.

Mutants lacking chlorophyll also have an association with chlorophyll degradation. Previous research demonstrated that *NYC1* encodes chlorophyll *b* reductase, which catalyzes the degradation of chlorophyll *b* to 7-hydroxymethyl chlorophyll *a*^30^. The degradation of chlorophyll *b* is suppressed in *NYC1* mutant plants, which remain green until just before death due to natural senescence^31^. Moreover, the *NOL* (NYC1-like) protein is closely related to *NYC1*, and chlorophyll *b* levels decreased drastically in *NOL* overexpressing plants^32,33^. In our study, two *NYC1/NOL* DEGs were detected, both of which were significantly up-regulated in the mutant compared with normal green leaves. Additionally, RT-qPCR analyses revealed that *NYC1* was significantly expressed in May and June in the mutant, indicating that they may be responsible for regulating chlorophyll degradation in the mutant. Combined with the results of physiological measurements, the content of chlorophyll *b*, but not that of chlorophyll *a*, was significantly lower in the mutant. Thus, we propose that higher expression levels of *NYC/NOL* genes may contribute to accelerated degradation of chlorophyll *b* to chlorophyll *a*, and eventually result in a mutant of *G. biloba* without chlorophyll.

### DEGs associated with chloroplast development and photosynthetic capacity

Normal development of chloroplasts in higher plants requires the coordination of chloroplast genes and nuclear genes. Alteration in the expression levels of either gene type may affect the biogenesis of normal chloroplasts. The resultant disruptions in chlorophyll metabolism and chloroplast assembly can lead to abnormal leaf color^8,23^. The *GLK* gene family encodes a pair of partially redundant nuclear TFs that are required for the expression of nuclear photosynthetic genes, and for chloroplast development^34^. It has been reported that *GLK* is involved in regulating chloroplast development in the leaves of *Z. mays*, *O. sativa* and *A. thaliana*^35–37^. Recent studies have also indicated that the ultrastructure of chloroplasts was disrupted, and the expression level of *GLK* gene was lower than that of the wild type in *L. indica* and *A. andraeanum* mutants^8,17^. In the present study, the expression level of *GbGLK* was very significantly decreased in the mutant compared with normal green leaves, similar to reports of other species^36^. RT-qPCR results further confirmed the importance of the *GbGLK* factor. Ftsz proteins, which play key roles in cell and organelle division, also regulate the division of plastids, such as chloroplasts^38^. In the leaf color mutant of *G. biloba*, reduced expression of *GbFtsz* together with a decrease in the number and size of chloroplasts, indicate that chloroplast division was likely defective. These gene effects were also strongly supported by ultrastructural differences between normal green leaves and mutants, indicating that leaf color variation in *G. biloba* was closely correlated with abnormal chloroplast development and division in leaves.

**Fig. 8:**
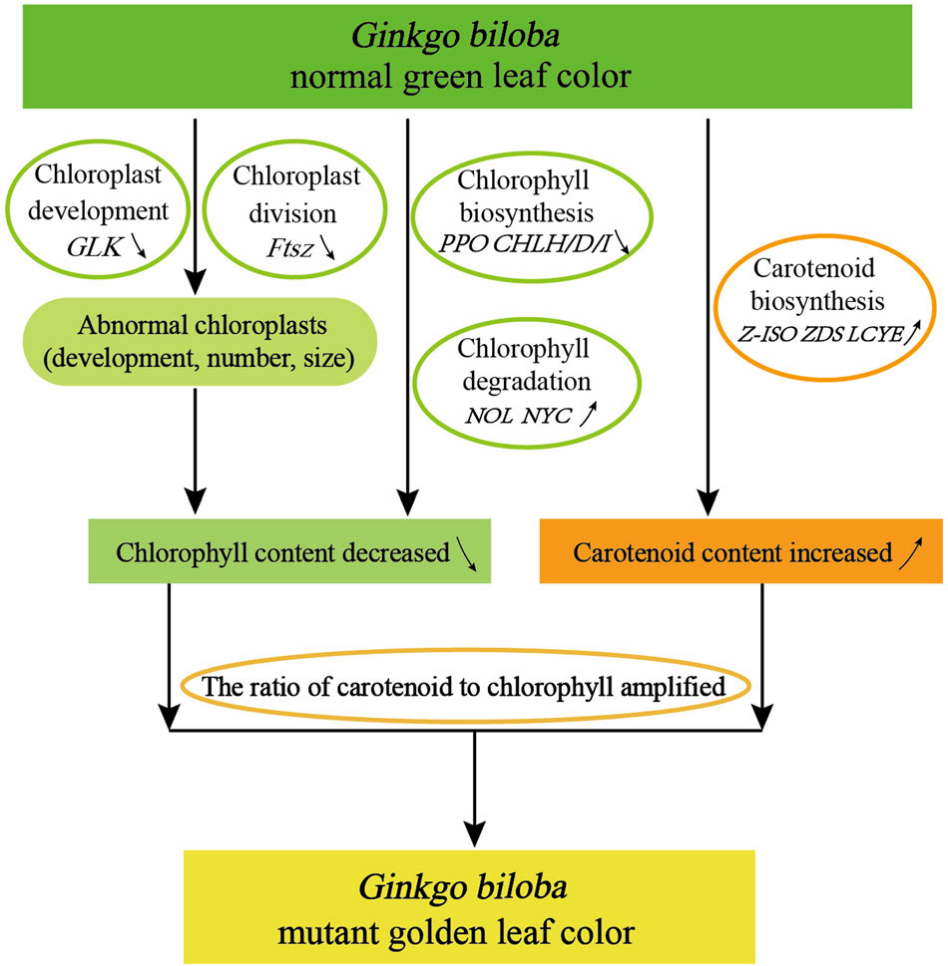
The proposed pathway of mutant leaf coloration in *G. biloba*

During photosynthesis, light energy is captured by pigments in LHC proteins, which then transfer the absorbed light energy to the reaction center complexes of PSI and PSII^39,40^. In a chlorophyll-deficient mutant of *A. thaliana*, LHC proteins were highly significantly decreased, or even completely absent, resulting in impaired grana stacking in the chloroplast^41^. The multi-protein and pigment complex PSII provides the high redox potential needed to oxidize water, and contains more than 20 subunits, including *PsbA*, *PsbD*, and *PsbP*^42^. PSI catalyzes light-driven electron transfer from luminal plastocyanin to stromal ferredoxin and consists of more than 10 subunits, including *PsaC* and *PsaL*. The *PsaL* subunit is responsible for the majority of interactions, and its mutants exhibit a slightly smaller functional size of the photosynthetic antenna and lower excitation levels^43^. In our study, nine DEGs were identified among the LHC gene family, and their expression levels were sharply reduced in the mutant, indicating a decrease in light-harvesting chlorophyll proteins relative to reaction center complexes. Furthermore, 18 DEGs related to the PSI reaction center, including *PsaD*, *PsaL*, and *PsaO*, and 15 DEGs associated with the PSII reaction center, such as *PsbP*, had reduced expression levels in the mutant. In particular, RT-qPCR results indicated that DEGs related to *LHCB* and *PSB* were expressed at significantly lower levels from May to July in the mutant. Considering the reduced photosynthetic capacity as in the mutant, as well as the extensive alteration in the expression of genes involved in photosynthesis, we conclude that DEGs in PSII might account for the reduced photosynthetic capacity in the *G. biloba* mutant (Fig. 8).

### DEGs related to carotenoid and flavonoid biosynthesis

Carotenoids are a family of pigments, ranging in color from yellow to red, which are involved in light harvesting and are indispensable for photoprotection under conditions of excess light^44,45^. Thus, impaired carotenoid biosynthesis can result in pleiotropic phenotypes^46,47^. In previous research on an *Oncidium* hybrid orchid, expression of the *PSY* gene was down-regulated in yellow leaves, whereas expression levels of other genes involved in carotenoid biosynthesis were enhanced^48^. In the present study, seven DEGs associated with carotenoid biosynthesis were identified; only one DEG encoding *PSY* was down-regulated, while other DEGs such as *Z-ISO*, *ZDS*, and *LCYE* were up-regulated in the mutant. The up-regulated *Z-ISO*, *ZDS* and LCYE as *PSY* downstream genes might offset the down-regulation of *PSY*. This enhanced expression of genes for carotenoid biosynthesis, and the increased carotenoid content in mutant leaves, suggests that carotenoids contribute to yellow coloration in *G. biloba* leaves.

Flavonoid biosynthetic metabolism includes other important pathways associated with coloration in plants. Flavonoids such as anthocyanins, flavonols and proanthocyanidins can be regulated by MYB, bHLH and WD TFs^49^. In *A. thaliana*, accumulation of flavonoids affected pigmentation in the tissues of the mutant^50^. In this study, 28 candidate genes involved in the flavonoid biosynthesis pathway were identified. Most of the expression of DEGs related to flavonoid biosynthesis, such as *GbPAL*, *GbCHS* and *GbANS*, was inhibited in the leaf color mutant. Additionally, 41 DEGs annotated to MYB/bHLH TFs were down-regulated in the mutant, aside from DEGs encoding AP2-EREBP, WRKY, C2H2 and the NAC family. Using differential expression analysis of the TFs identified, we found that MYB39 and bHLH25 TFs play important roles in the control of flavonoid biosynthesis and may influence leaf coloration. Furthermore, RT-qPCR analysis revealed that *PAL*, *CHS*, *MYB39* and *bHLH25* were expressed at significantly lower levels from May to July in the mutant compared to normal green leaves. At the physiological level, the total flavonoid content results showed less accumulation of flavonoids in the mutant than in the green leaves. Although the flavonoids contain anthocyanins, flavones and flavonols, which contribute to leaf coloration, the lower flavonoid accumulation in the *G. biloba* mutant suggests that flavonoids might not be essential to the yellow coloration seen in this species.

## Conclusions

In this study, we investigated differences in coloration between normal green leaves and gold-colored mutant leaves of *G. biloba*. Lower chlorophyll contents and abnormal ultrastructural characteristics of chloroplasts were observed in the mutant leaves. Transcriptional sequence analysis identified 116 DEGs and 46 TFs involved in chlorophyll metabolism, chloroplast development and division, photosynthesis and other pigment biosynthesis. Furthermore, RT-qPCR experiments verified that those DEGs were differentially expressed between the normal green leaves and the leaf color mutant. Changes in the expression of genes related to chloroplast development, chlorophyll biosynthesis and degradation led to less chlorophyll accumulation in the mutant leaves. Enhanced carotenoid biosynthesis contributed to higher carotenoid contents especially lutein. The ratio of carotenoids to chlorophylls in the mutant was amplified by decreased chlorophylls and increased carotenoids, which might have a close connection with the golden leaf coloration. Our findings provide insights into the molecular mechanism underlying the yellowing phenotype in plants.

## Data archive statement

All clean and processed transcriptomic sequence data used in this research have been deposited in the Gene Expression Omnibus (GEO) under the accession number GSE103827.

## Electronic supplementary material

Supplementary material

## References

[1] Feild TS, Lee DW, Holbrook NM (2001). Why leaves turn red in autumn. The role of anthocyanins in senescing leaves of red-osier dogwood. Plant Physiol..

[2] Nagata N, Tanaka R, Satoh S, Tanaka A (2005). Identification of a vinyl reductase gene for chlorophyll synthesis in A*rabidopsis thaliana* and implications for the evolution of prochlorococcus species. Plant Cell.

[3] Adhikari ND (2011). GUN4-porphyrin complexes bind the ChlH/GUN5 subunit of Mg-Chelatase and promote chlorophyll biosynthesis in A*rabidopsis*. Plant Cell.

[4] Wu Z (2007). A chlorophyll-deficient rice mutant with impaired chlorophyllide esterification in chlorophyll biosynthesis. Plant Physiol..

[5] Wang L (2014). Biochemical and transcriptome analyses of a novel chlorophyll-deficient chlorina tea plant cultivar. BMC Plant Biol..

[6] Zhu G (2015). Transcriptome characterization of *Cymbidium sinense* ‘Dharma’ using 454 pyrosequencing and its application in the identification of genes associated with leaf color variation. PLoS One.

[7] Dong H (2013). A rice v*irescent-yellow leaf* mutant reveals new insights into the role and assembly of plastid caseinolytic protease in higher plants. Plant Physiol..

[8] Li Y (2015). Comprehensive transcriptome analysis discovers novel candidate genes related to leaf color in a L*agerstroemia indica* yellow leaf mutant. Genes Genom..

[9] Li CF (2016). Biochemical and transcriptomic analyses reveal different metabolite biosynthesis profiles among three color and developmental stages in Anji Baicha (C*amellia sinensis)*. BMC Plant Biol..

[10] Wang L (2011). An embryological study and systematic significance of the primitive gymnosperm *Ginkgo biloba*. J. Syst. Evol..

[11] Matile P, Flach BM, Eller BM (1992). Autumn leaves of *Ginkgo biloba* L.: optical properties, pigments and optical brighteners. Plant Biol..

[12] Liu X (2016). Comparative proteomic and physiological analysis reveals the variation mechanisms of leaf coloration and carbon fixation in a Xantha mutant of *Ginkgo biloba* L. Int J. Mol. Sci..

[13] Benjamini Y, Hochberg Y (1995). Controlling the false discovery rate: a practical and powerful approach to multiple testing. J. R. Stat. Soc. Series B Stat Methodol.

[14] Livak KJ, Schmittgen TD (2001). Analysis of relative gene expression data using real-time quantitative PCR and the 2^—△△Ct^ method. Methods.

[15] Eckhardt U, Grimm B, Hörtensteiner S (2004). Recent advances in chlorophyll biosynthesis and breakdown in higher plants. Plant Mol. Biol..

[16] Hörtensteiner S (2013). Update on the biochemistry of chlorophyll breakdown. Plant Mol. Biol..

[17] Yang Y (2015). Phenotype and transcriptome analysis reveals chloroplast development and pigment biosynthesis together influenced the leaf color formation in mutants of *Anthurium andraeanum ‘*Sonate’. Front. Plant Sci..

[18] Lonosky PM (2004). A proteomic analysis of maize chloroplast biogenesis. Plant Physiol..

[19] Li YH (2012). Morphological structure and genetic mapping of new leaf-color mutant gene in rice (*Oryza sativa*). Rice Sci..

[20] Maekawa S (2015). Pale-green phenotype of atl31atl6 double mutant leaves is caused by disruption of 5-aminolevulinic acid biosynthesis in *Arabidopsis thaliana*. PLoS One.

[21] Wu ZM, Zhang X, Wang JL, Wan JM (2014). Leaf chloroplast ultrastructure and photosynthetic properties of a chlorophyll-deficient mutant of rice. Photosynthetica.

[22] Yoo SC (2009). Ricev*irescent3* and *stripe1* encoding the large and small subunits of ribonucleotide reductase are required for chloroplast biogenesis during early leaf development. Plant Physiol..

[23] Yang HY (2015). Identification of genes involved in spontaneous leaf color variation in *Pseudosasa japonica*. Genet. Mol. Res..

[24] Seo TS (2004). Photocleavable fluorescent nucleotides for DNA sequencing on a chip constructed by site-specific coupling chemistry. Proc. Natl. Acad. Sci. USA.

[25] Wang Z, Gerstein M, Snyder M (2009). RNA-Seq: a revolutionary tool for transcriptomics. Nat. Rev. Genet..

[26] Fromme P (2003). Structure and function of photosystem I: interaction with its soluble electron carriers and external antenna systems. FEBS Lett..

[27] Meier S (2011). A transcriptional analysis of carotenoid, chlorophyll and plastidial isoprenoid biosynthesis genes during development and osmotic stress responses in *Arabidopsis thaliana*. BMC Syst. Biol..

[28] Kumar AM, Söll D (2000). Antisense *HEMA1* RNA expression inhibits heme and chlorophyll biosynthesis in *Arabidopsis*. Plant Physiol..

[29] Koch M (2004). Crystal structure of protoporphyrinogen IX oxidase: a key enzyme in haem and chlorophyll biosynthesis. EMBO J..

[30] Lai B (2015). Transcriptomic analysis of *Litchi chinensis* pericarp during maturation with a focus on chlorophyll degradation and flavonoid biosynthesis. BMC Genom..

[31] Kusaba M (2007). Rice *NON-YELLOW COLORING1* is involved in light-harvesting complex II and grana degradation during leaf senescence. Plant Cell.

[32] Sato Y (2009). Two short-chain dehydrogenase/reductases, NON-YELLOW COLORING 1 and NYC1- LIKE, are required for chlorophyll *b* and light-harvesting complex II degradation during senescence in rice. Plant J..

[33] Jia T, Ito H, Tanaka A (2015). The Chlorophyll *b*, reductase NOL participates in regulating the antenna size of photosystem II in *Arabidopsis thaliana*. *Procedia*. Chemistry.

[34] Waters MT, Moylan EC, Langdale JA (2008). GLK transcription factors regulate chloroplast development in a cell-autonomous manner. Plant. J..

[35] Hall LN (1998). GOLDEN 2: A novel transcriptional regulator of cellular differentiation in the maize leaf. Plant Cell.

[36] Fitter DW (2002). *GLK* gene pairs regulate chloroplast development in diverse plant species. Plant J..

[37] Waters MT (2009). GLK transcription factors coordinate expression of the photosynthetic apparatus in *Arabidopsis*. Plant Cell.

[38] Schmitz AJ, Glynn JM, Olson BJ, Stokes KD, Osteryoung KW (2009). *Arabidopsis* FtsZ2-1 and FtsZ2-2 are functionally redundant, but FtsZ-based plastid division is not essential for chloroplast partitioning or plant growth and development. Mol. Plant.

[39] Goral T (2012). Light-harvesting antenna composition controls the macrostructure and dynamics of thylakoid membranes in *Arabidopsis*. Plant J..

[40] Zhao X (2016). Non-photochemical quenching plays a key role in light acclimation of rice plants differing in leaf color. Front. Plant Sci..

[41] Kim EH (2009). The multiple roles of light-harvesting chlorophyll a/b-protein complexes define structure and optimize function of *Arabidopsis* chloroplasts: a study using two chlorophyll *b*-less mutants. BBA.

[42] Shi LX (2012). Photosystem II, a growing complex: updates on newly discovered components and low molecular mass proteins. BBA.

[43] Klodawska K (2013). Morphological and physiological characterization of the delta-psaL mutant of *cyanobacterium Synechocystis*. Polyhedron.

[44] Cazzonelli CI, Pogson BJ (2010). Source to sink: regulation of carotenoid biosynthesis in plants. Trends Plant. Sci..

[45] Song L (2017). Molecular link between leaf coloration and gene expression of flavonoid and carotenoid biosynthesis in *Camellia sinensis* cultivar ‘Huangjinya’. Front Plant Sci..

[46] Dong H (2007). The *Arabidopsis Spontaneous Cell Death1* gene, encoding a zeta-carotene desaturase essential for carotenoid biosynthesis, is involved in chloroplast development, photoprotection and retrograde signalling. Cell Res..

[47] Yuan H (2015). Carotenoid metabolism and regulation in horticultural crops. Hortic. Res..

[48] Liu JX (2014). RNA interference-based gene silencing of *phytoene synthase* impairs growth, carotenoids, and plastid phenotype in *Oncidium hybrid* orchid. SpringerPlus.

[49] Xu WJ, Dubos C, Lepiniec L (2015). Transcriptional control of flavonoid biosynthesis by MYB-bHLH-WDR complexes. Trends Plant Sci..

[50] Appelhagen I (2011). Leucoanthocyanidin dioxygenase in *Arabidopsis thaliana*: characterization of mutant alleles and regulation by MYB-BHLH-TTG1 transcription factor complexes. Gene.

