# Publisher Correction: Cytological, physiological, and transcriptomic analyses of golden leaf coloration in *Ginkgo biloba* L

**DOI:** 10.1038/s41438-018-0039-9

**Published:** 2018-06-01

**Authors:** Wei-xing Li, Shun-bo Yang, Zhaogeng Lu, Zhi-chong He, Yun-ling Ye, Bei-bei Zhao, Li Wang, Biao Jin

**Affiliations:** 1 College of Horticulture and Plant Protection, Yangzhou University, 225009 Yangzhou, China; 2 College of Resource and Environment, Xizang Agriculture and Animal Husbandry College, 860000 Tibet, China

**Correction to**: *Horticulture Research* (2018) **5**: 12; 10.1038/s41438-018-0015-4; Article published online 1 March 2018.

In the original publication of this article [1] not all authors were listed correctly. In this correction article the full author list is given. The original publication has been corrected.
