# Supplementary material for: Cytological, physiological, and transcriptomic analyses of golden leaf coloration in Ginkgo biloba L
Source: Hortic Res. 2018 Mar 1;5:12. doi: 10.1038/s41438-018-0015-4 (PMC5830439; doi:10.1038/s41438-018-0015-4)
Supplement: Supplementary file 1 — Supplementary material [file 41438_2018_15_MOESM1_ESM.doc]

**Figure S1**

**
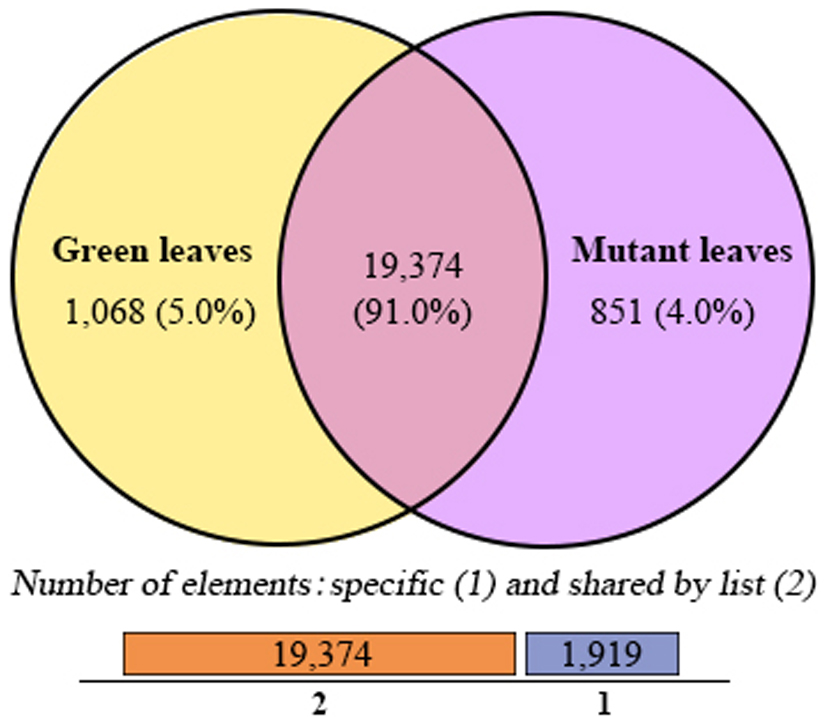
**

**Figure S2**

**
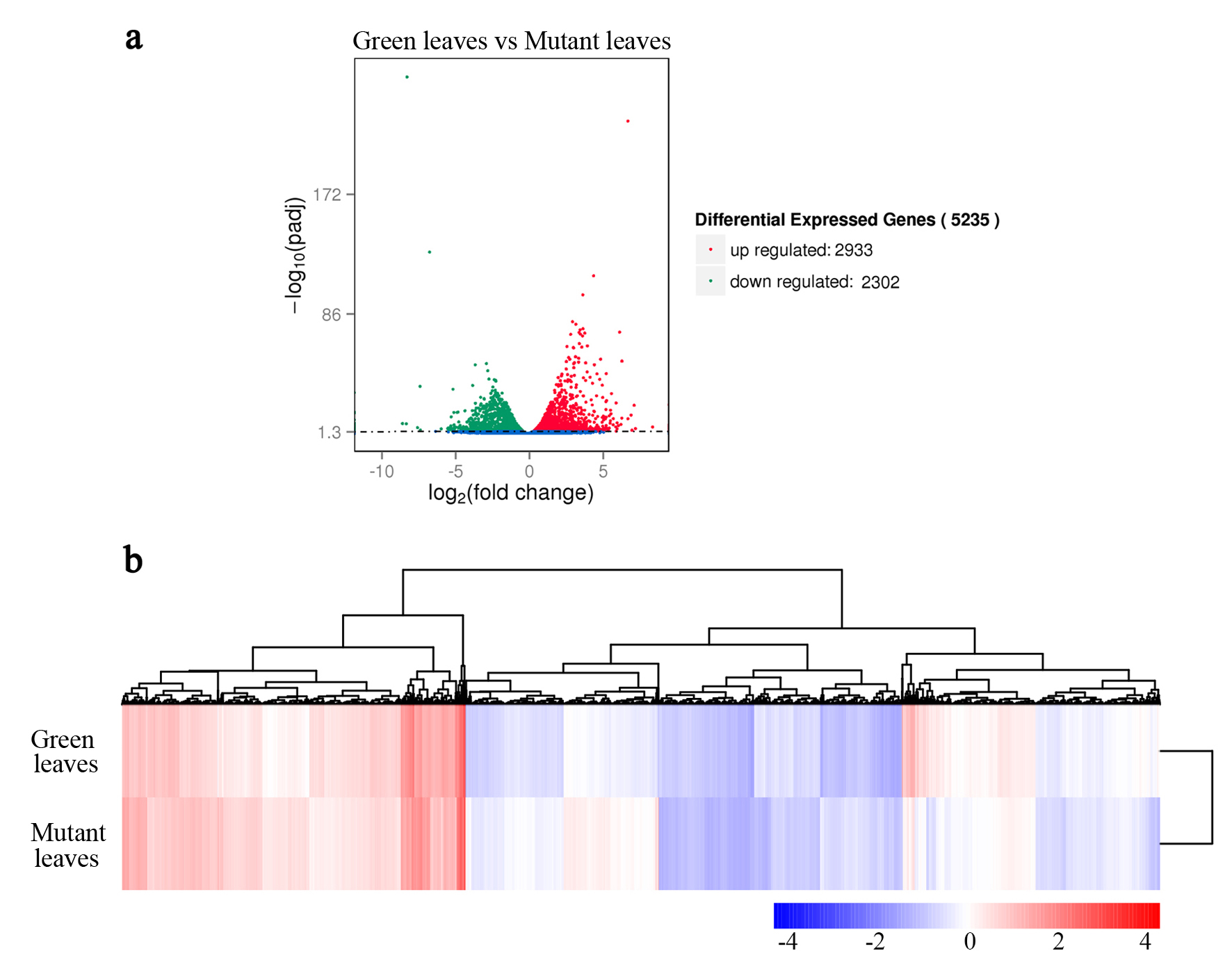
**

**Figure S3**

**
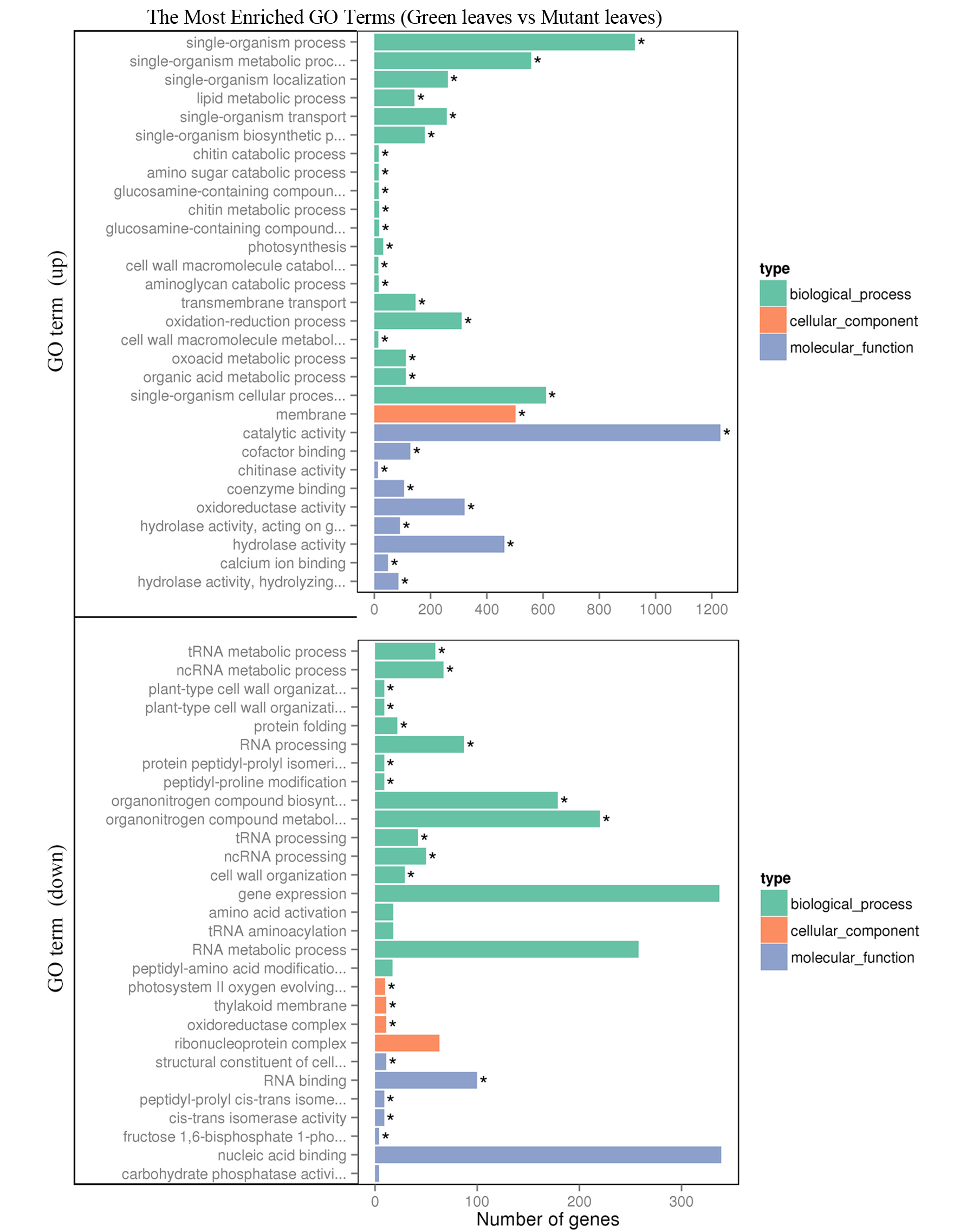
**

**Figure S4**

**
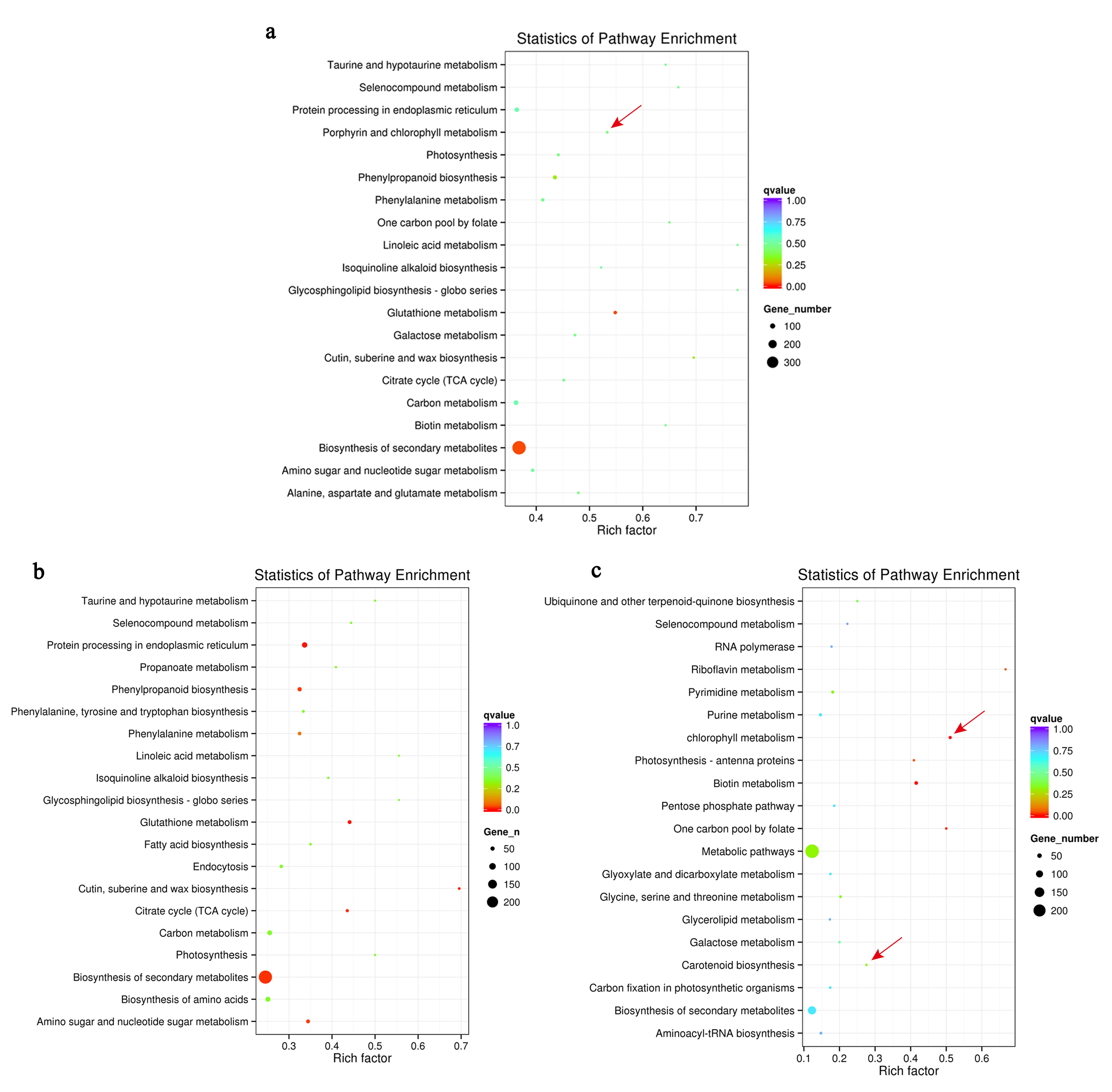
**

**Figure S5**

**
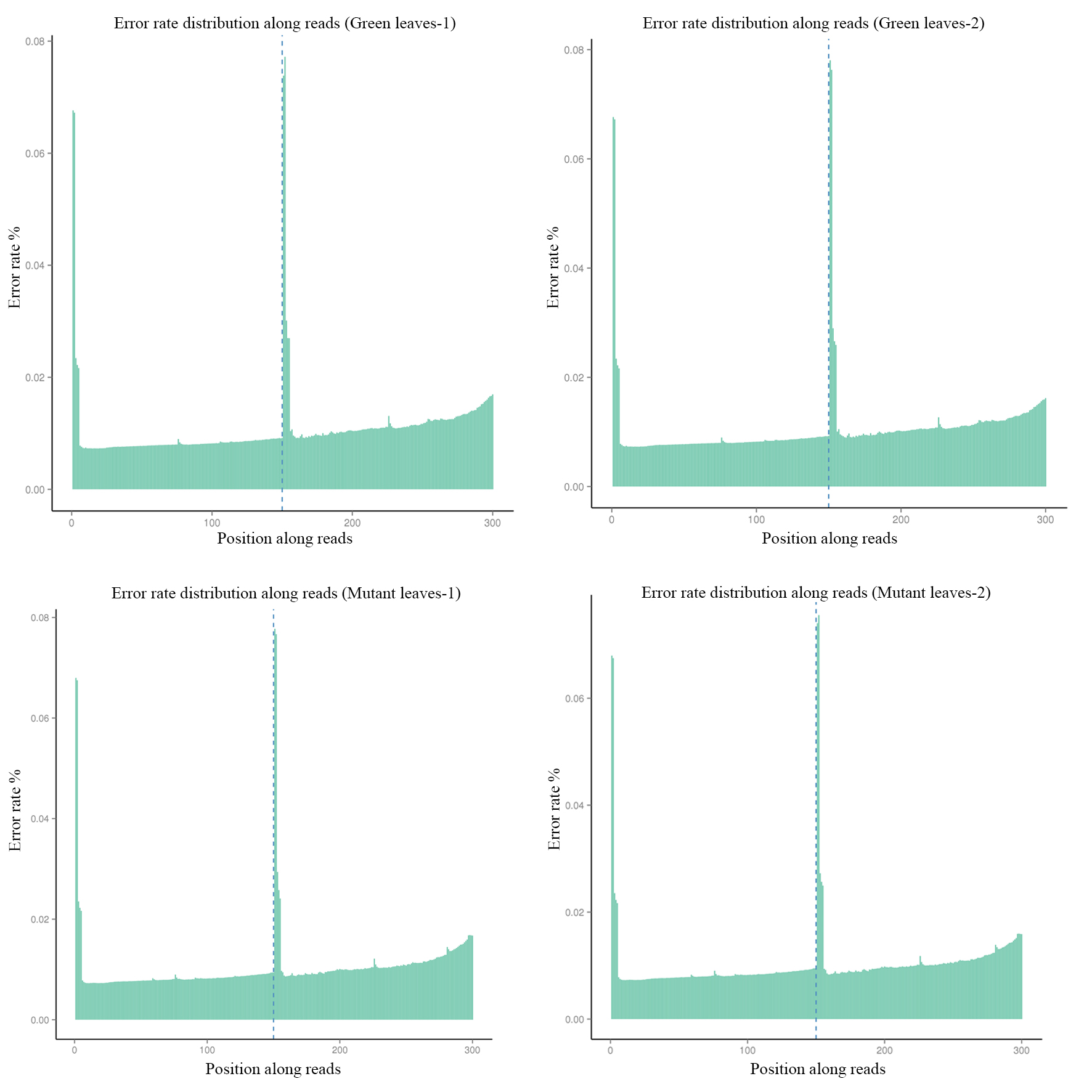
**

**Figure S6**

**
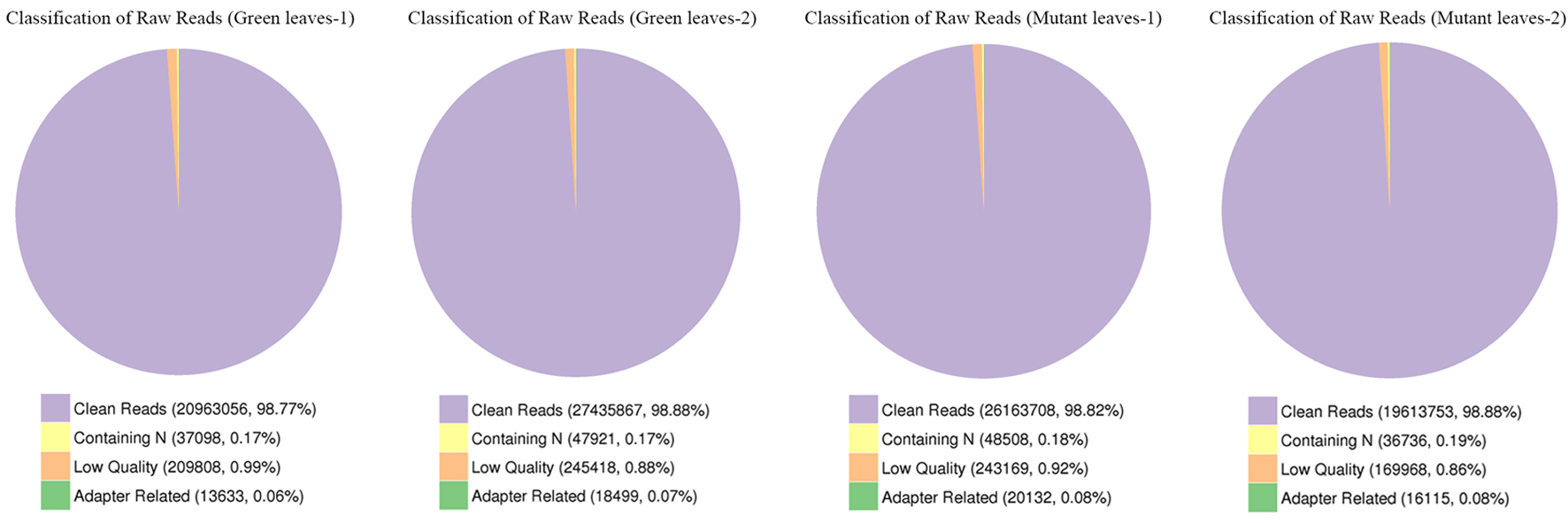
**

**Figure S7**

**
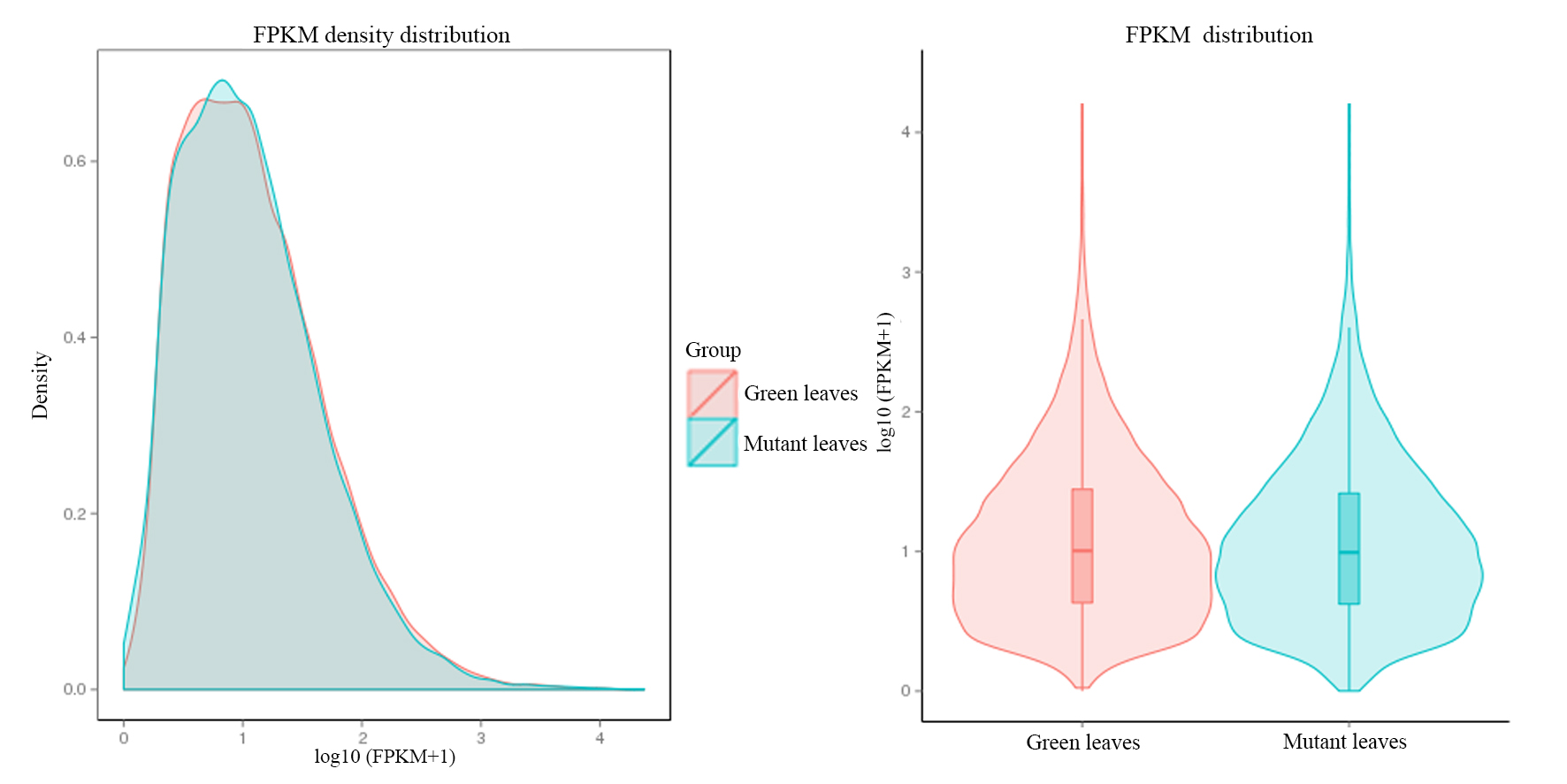
**

**Figure S8**

**
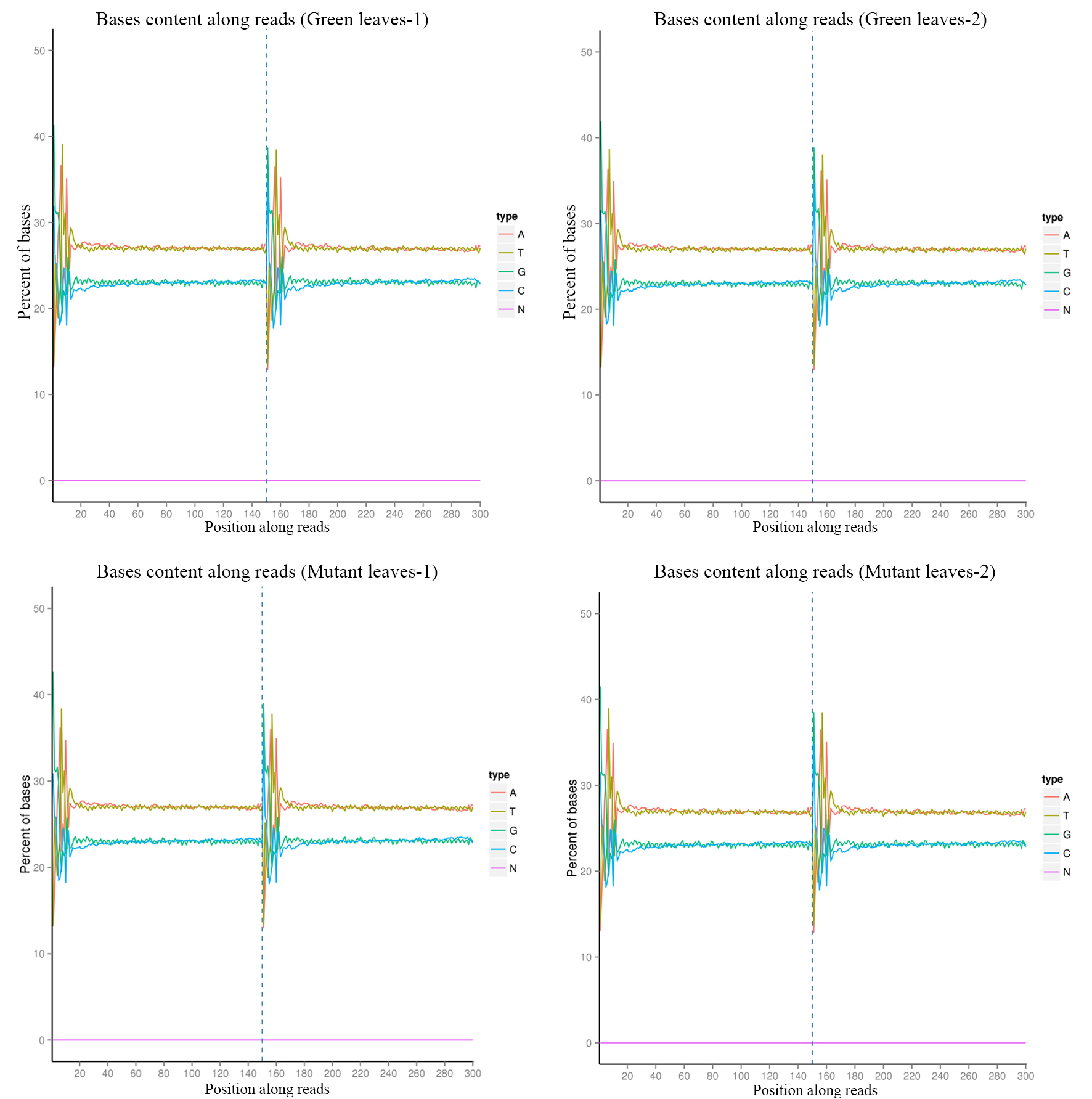
**

**Figure S9**

**
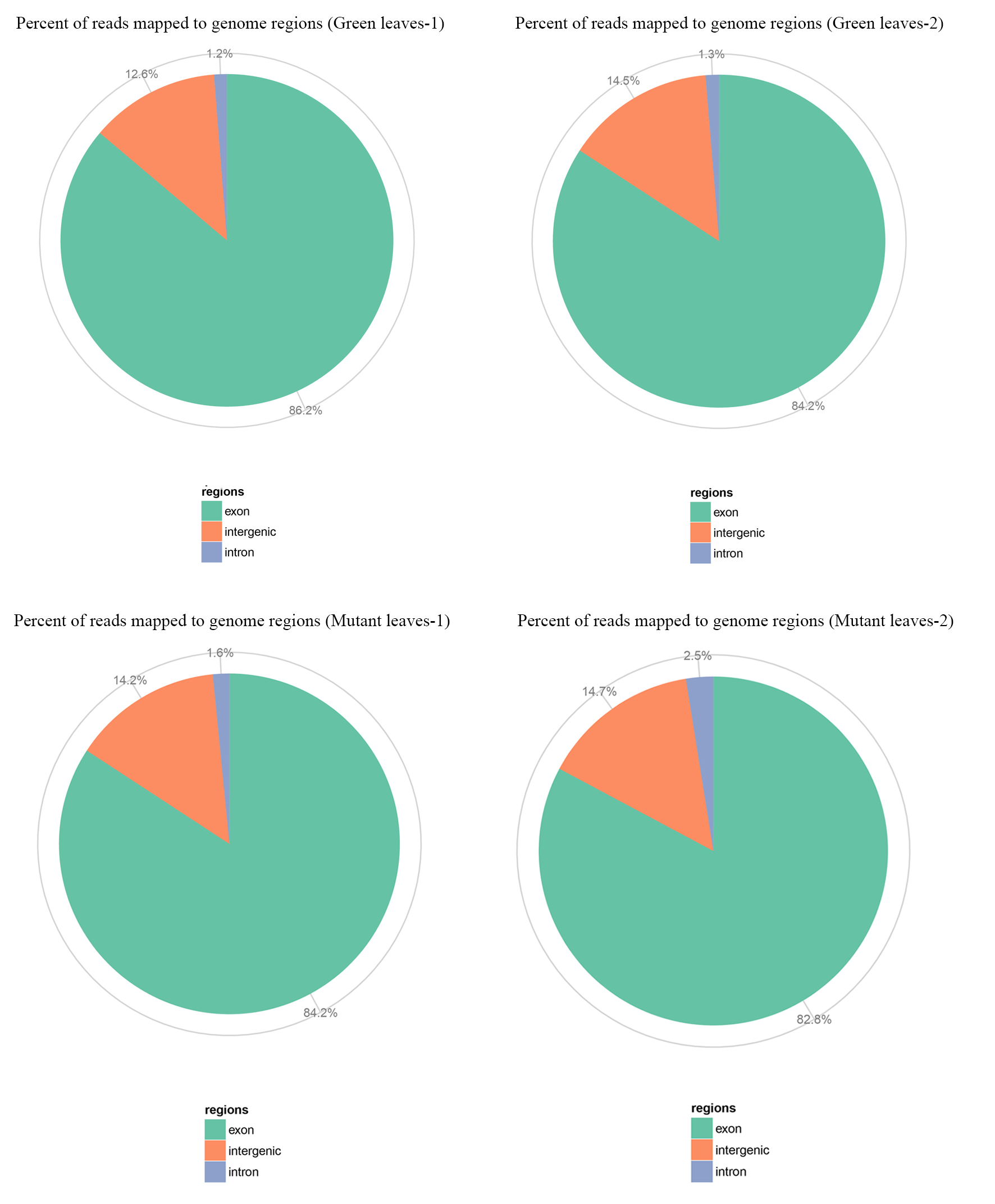
**

**Figure S10**

**
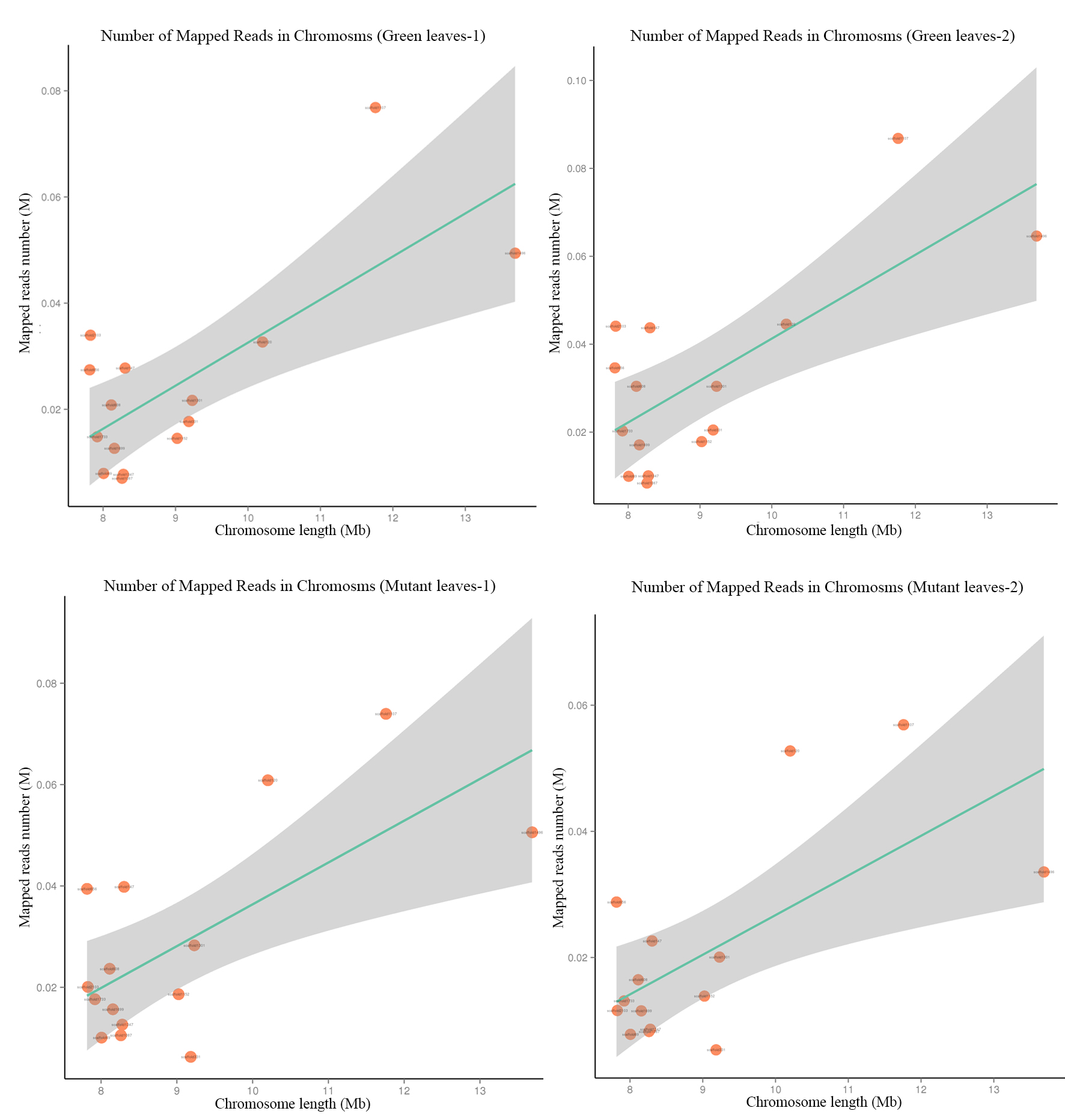
**

**Figure S11**

**
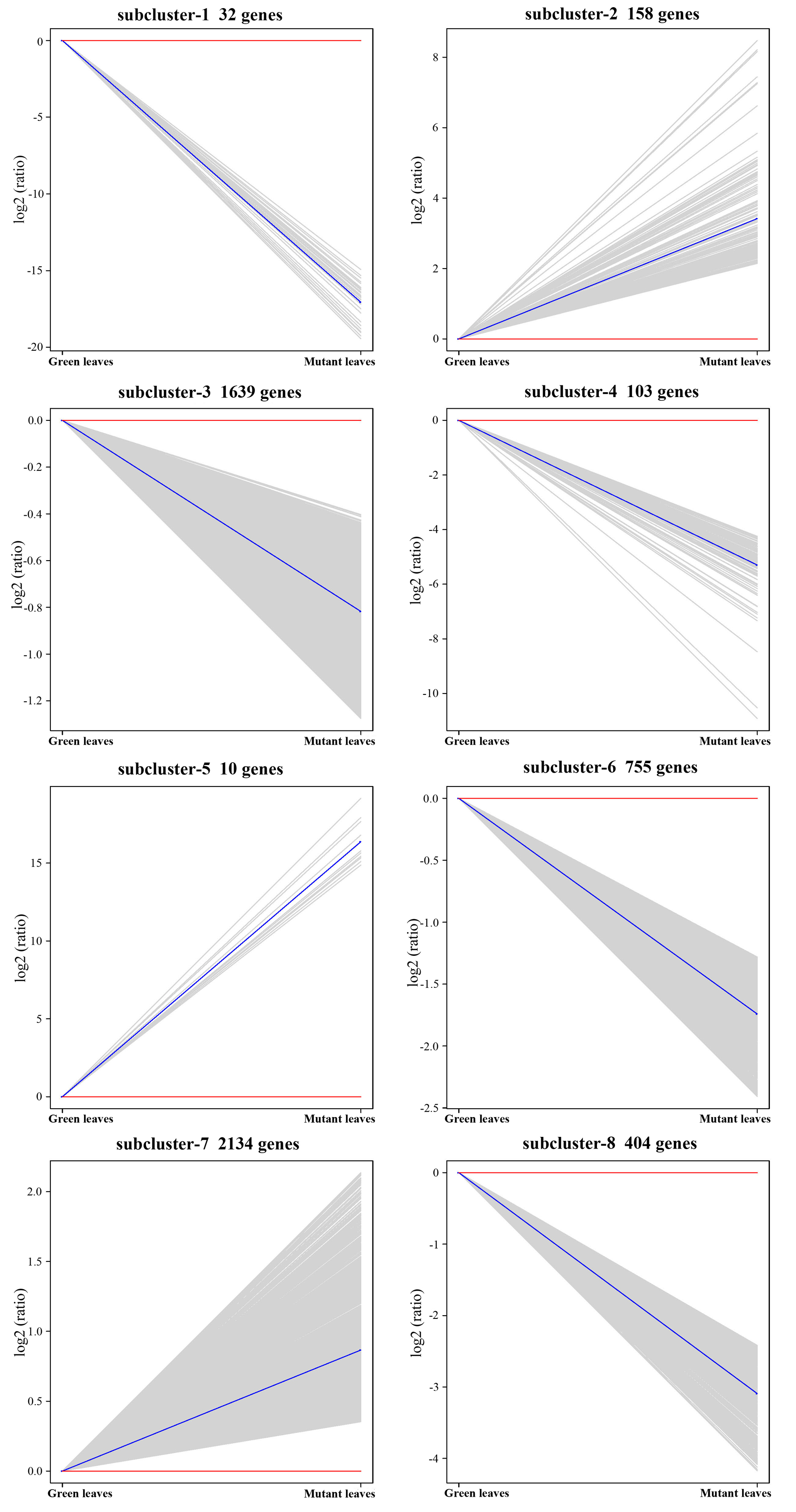
**

**Table S1**

| **Sample name** | **Green leaf-1** | **Green leaf-2** | **Mutant-1** | **Mutant-2** |
| --- | --- | --- | --- | --- |
| Raw reads | 42447190 | 55495410 | 52951034 | 39673144 |
| Clean reads | 41926112 | 54871734 | 52327416 | 39227506 |
| Clean bases | 6.21G | 8.12G | 7.75G | 5.81G |
| Error rate(%) | 0.01 | 0.01 | 0.01 | 0.01 |
| Q20(%) | 97.93 | 98.03 | 98.12 | 98.20 |
| Q30(%) | 95.30 | 95.48 | 95.68 | 95.85 |
| GC content(%) | 46.25 | 46.21 | 46.32 | 46.42 |
| Total mapped | 39479380 (94.16%) | 51720246 (94.26%) | 49547505 (94.69%) | 37014921 (94.36%) |
| Multiple mapped | 2322259 (5.54%) | 3035965 (5.53%) | 2706406 (5.17%) | 2195146 (5.6%) |
| Uniquely mapped | 37157121 (88.63%) | 48684281 (88.72%) | 46841099 (89.52%) | 34819775 (88.76%) |

**Table S2**

| **Function** | **Gene ID** | **TF family** | **Expression level in mutant** | **Annotation** |
| --- | --- | --- | --- | --- |
| Chlorophyll biosynthesis | Gb_08295 | HEMA | Up-regulated | Glutamyl-tRNA reductase |
|  | Gb_37725 | HEMA | Up-regulated | Glutamyl-tRNA reductase |
|  | Gb_13789 | HEMB | Up-regulated | Delta-aminolevulinic acid dehydratase |
|  | Gb_08885 | HEMC | Up-regulated | Porphobilinogen deaminase |
|  | Gb_19378 | HEME | Up-regulated | Uroporphyrinogen decarboxylase |
|  | Gb_37977 | HEMF | Up-regulated | Coproporphyrinogen-III oxidase |
|  | Gb_20294 | HEMF | Up-regulated | Coproporphyrinogen-III oxidase |
|  | Gb_09338 | PPO | Down-regulated | Protoporphyrinogen oxidase |
|  | Gb_09336 | PPO | Down-regulated | Protoporphyrinogen oxidase |
|  | Gb_09333 | PPO | Up-regulated | Protoporphyrinogen oxidase |
|  | Gb_09332 | PPO | Down-regulated | Protoporphyrinogen oxidase |
|  | Gb_09340 | PPO | Down-regulated | Protoporphyrinogen oxidase |
|  | Gb_06427 | CHLH | Down-regulated | Magnesium-chelatase subunit ChlH |
|  | Gb_04254 | CHLD | Down-regulated | Magnesium-chelatase subunit ChlD |
|  | Gb_27010 | ChlI | Down-regulated | Magnesium-chelatase subunit ChlI |
|  | Gb_19731 | CHLM | Down-regulated | Magnesium protoporphyrin IX methyltransferase |
|  | Gb_35840 | CRD | Down-regulated | Magnesium-protoporphyrin IX monomethyl ester [oxidative] cyclase |
|  | Gb_24083 | CHLG | Down-regulated | Chlorophyll synthase |
|  | Gb_34355 | HCAR | Up-regulated | 7-hydroxymethyl chlorophyll a reductase |
|  | Gb_29139 | NYC | Up-regulated | Chlorophyll(ide) b reductase NYC1 |
|  | Gb_38508 | NOL | Up-regulated | Chlorophyll(ide) b reductase NOL |
|  | Gb_04951 | PPH | Up-regulated | Pheophytinase, chloroplastic |
|  | Gb_20648 | RCCR | Up-regulated | Red chlorophyll catabolite reductase |
| Chloroplast development | Gb_05662 | GLK | Down-regulated | Transcription activator GLK1 |
| Chloroplast division | Gb_17194 | Ftsz | Down-regulated | Cell division protein FtsZ homolog 1 |
|  | Gb_27796 | Ftsz | Down-regulated | Cell division protein FtsZ homolog 2 |
|  | Gb_29948 | Ftsz | Up-regulated | Cell division protein FtsZ homolog 2 |

**Table S3**

| **Function** | **Gene ID** | **TF family** | **Expression level in mutant** | **Annotation** |
| --- | --- | --- | --- | --- |
| Carotenoid biosynthesis | Gb_27208 | PSY | Down-regulated | Phytoene synthase |
|  | Gb_24507 | ZISO | Up-regulated | 15-cis-zeta-carotene isomerase |
|  | Gb_09570 | ZDS | Up-regulated | Zeta-carotene desaturase |
|  | Gb_26868 | LCYE | Up-regulated | Lycopene epsilon cyclase |
|  | Gb_27593 | VDE | Up-regulated | Violaxanthin de-epoxidase |
|  | Gb_24102 | ZEP | Up-regulated | Zeaxanthin epoxidase |
|  | Gb_32510 | ZEP | Up-regulated | Zeaxanthin epoxidase |
| Flavonoid biosynthesis | Gb_01672 | PAL | Down-regulated | Phenylalanine ammonia-lyase |
|  | Gb_10949 | PAL | Up-regulated | Phenylalanine ammonia-lyase |
|  | Gb_25608 | PAL | Down-regulated | Phenylalanine ammonia-lyase |
|  | Gb_40571 | 4CL | Down-regulated | 4-coumarate--CoA ligase |
|  | Gb_19002 | CHS | Down-regulated | Chalcone synthase |
|  | Gb_20355 | CHS | Down-regulated | Chalcone synthase |
|  | Gb_19001 | CHS | Down-regulated | Chalcone synthase |
|  | Gb_24242 | DFR | Down-regulated | Dihydroflavonol-4-reductase |
|  | Gb_09087 | DFR | Up-regulated | Dihydroflavonol-4-reductase |
|  | Gb_26470 | DFR | Down-regulated | Dihydroflavonol-4-reductase |
|  | Gb_21870 | ANS | Down-regulated | Leucoanthocyanidin dioxygenase |
|  | Gb_33402 | ANS | Down-regulated | Leucoanthocyanidin dioxygenase |
|  | Gb_21868 | ANS | Down-regulated | Leucoanthocyanidin dioxygenase |
|  | Gb_21869 | ANS | Down-regulated | Leucoanthocyanidin dioxygenase |
|  | Gb_21859 | ANS | Down-regulated | Leucoanthocyanidin dioxygenase |
|  | Gb_11130 | ANS | Up-regulated | Leucoanthocyanidin dioxygenase |
|  | Gb_35183 | UFGT | Up-regulated | Anthocyanidin 3-O-glucosyltransferase |
|  | Gb_03245 | UFGT | Down-regulated | Anthocyanidin 3-O-glucosyltransferase |

**Table S4**

| **Gene ID** | **Forward primer (5’–3’)** | **Reverse primer (3’–5’)** |
| --- | --- | --- |
| Gb_08295 | CCAGACCTTCATTGCTCAGGT | ATCATCATCTCCGCCAGCTT |
| Gb_09338 | AAGGCAAGCTGAGGCCAGT | CCTCAACAGACTCCTCTCGACTC |
| Gb_29139 | GGCCACGGAATGTTGTCATA | TGCGTCACAGTTGTCTGCAC |
| Gb_05662 | TCCTTGCTCGTGAAGCAGAA | TTGGAGGATTGCCACTGTGT |
| Gb_05853 | TGCACGCACAGAGCATACTG | GGTCCTCCGGCAATTCTGTA |
| Gb_35125 | TGCAGCCTTGGTACTTGGTG | TGCAGCAAGAGAGCCTGAAC |
| Gb_15656 | CCAGGTCGAATATGCCATTG | TTGGTTCCAGCAGTGGTGAC |
| Gb_05115 | CTTCCTCTCAATGCCGGTCT | TGCTCTTCCTGCAAGGTGAA |
| Gb_04842 | GACATTGGCGGTTCTCTTCC | CGACGCCTCTCATATCCTGTT |
| Gb_27208 | TGAAGTCTGTGCTGAGTATGCGAAG | AAGTTCATCAGTCCTCCATAGATTGCC |
| Gb_24507 | AAGCCAATAAGGTCGGAAGTTCTGC | ACACTGCTGTTACCACGCCAAG |
| Gb_09570 | TCATCAACGAAGGTGGACGAATTGG | GGACTCGTGGCAAGAGCAACTG |
| Gb_26868 | AGCGGTTGCGACTGATACTTGTG | TGCGTATTCAACTTCAGTTCCACCAG |
| Gb_01672 | GCAGAAGTTGACCAGACCAGAGATG | CTCATACAGCGGATACGAACGACAG |
| Gb_19002 | CGAATTACCAACAGCGAGCACAAG | TCTCCTTCAGTATCTCCTCCGTCAAG |
